# Optimizing clinical prediction model for new-onset atrial fibrillation in critically ill patient: Based on machine learning

**DOI:** 10.1371/journal.pone.0331857

**Published:** 2025-09-11

**Authors:** Da-cheng Wang, Xin-yuan Zhang, Xiao-huan Zhuang, Yan Zhuang

**Affiliations:** 1 Department of Critical Care Medicine, Affiliated Hospital of Nanjing University of Chinese Medicine, Nanjing, Jiangsu, China; 2 Department of Critical Care Medicine, Jiangsu Province Hospital of Chinese Medicine, Nanjing, Jiangsu, China; Azienda Ospedaliero Universitaria Careggi, ITALY

## Abstract

**Background:**

New-onset atrial fibrillation (NOAF) increases the risk of embolism and sudden death in critically ill patients; however, limited data exist attempting to identify modifiable risk factors and predict the incidence of NOAF. We aimed to investigate the risk factors for NOAF and develop an optimized clinical prediction model based on machine learning algorithms.

**Materials and methods:**

Data from patients admitted to the intensive care unit (ICU) of the Affiliated Hospital of Nanjing University of Chinese Medicine from August 2019 to January 2022 were retrospectively analyzed. LASSO regression and Random Forest (RF) algorithms were used to screen predictive variables. Logistic Regression, RF, Gradient Boosting and Support Vector Machine models were constructed to evaluate the recognition ability of different machine learning algorithms. The confusion matrix and calibration curve were used to assess the degree of accuracy of the four models. Decision curve analysis (DCA) was conducted to evaluate the utility of the model in decision-making. The net reclassification index (NRI) and integrated discrimination improvement (IDI) were also calculated to evaluate the performance of the models. The learning curves of the four models were plotted to evaluate the precision of different models. The SHapley Additive exPlanations (SHAP) was used to explain the supreme-performing model.

**Results:**

In total, 417 patients were enrolled in the study, and 333 patients were allocated to the training group and 84 to the validation group. The baseline characteristic distributions were similar between the two groups. Age, heart rate, mean arterial pressure, activated partial thromboplastin time, and brain natriuretic peptide were revealed as independent predictors of NOAF by LASSO regression and the RF algorithm. The RF model had the best performance, with the area under the receiver operator characteristic curve (AUROC) of 0.758, the area under the precision-recall curve (AUPRC) of 0.524, and accuracy of 0.735 in the training set, paralleled by AUROC of 0.796, AUPRC of 0.686, and accuracy of 0.702 in the validation set. The confusion matrix and calibration curves showed that RF had the best performance. DCAs also showed that the RF model provided the highest net benefit in the clinical setting. The NRI results showed that the RF significantly improved reclassification ability compared to the baseline model (NRI = 0.38). The IDI results further demonstrated a moderate improvement in discrimination ability for the RF (IDI = 0.033) compared to the baseline. The learning curves revealed that RF also showed superior performance. SHAP could be used visualized individual NOAF risk predicted by the model.

**Conclusions:**

The RF model exhibited the best performance in predicting NOAF in critically ill patients and has the potential to help clinicians identify high-risk patients and guide clinical decision making.

## Introduction

Atrial fibrillation (AF) is one of the commonest arrhythmias in intensive care unit (ICU), which leads to cardiac insufficiency, increases the risk of embolism and sudden death [1,2]. These adverse effects are especially pronounced in patients with new-onset atrial fibrillation (NOAF) [3]. NOAF is not only an indicator of the severity of the disease, but also an indicator of poor prognosis, and its prediction efficiency is no less than the other organ dysfunction [4]. Studies found that the incidence of NOAF in general ICU reached 4.5–29.5%, especially higher in sepsis patients [5,6]. Unlike AF seen in the general population, NOAF occurred in ICU was usually considered to be a result caused by pathophysiology and therapeutic measures of critical illness, including electrolyte disturbances, inflammatory reaction, or inotropes and vasopressors, which can induce arrhythmia [7,8]. Many traditional cardiovascular risk factors were thought to be linked with NOAF, but some of which are nonmodifiable [9]. Identifying modifiable risk factors and giving targeted interventions could reduce the incidence of NOAF, unfortunately the explicit predictors of NOAF in critical patients were limited in the literature [10].

The clinical prediction model is a practical disease prediction method in clinical research, which analyzes risk factors associated with the disease to predict complications or outcomes and can be used for individualized assessment of patient-related risks [11,12]. Machine learning techniques have demonstrated immense potential in predicting the probability of an event occurrence, which could be used to construct prediction models [13,14]. In medical prediction tasks, different machine learning models offer unique characteristics and applicability. Logistic regression (LR) is widely used in NOAF prediction due to its interpretability [15–17]. In consideration of complexities of data, such as high-dimensional and nonlinear classification problems, other prediction models such as random forest (RF), Gradient Boosting (GB) and support vector machine (SVC) were also used to predict AF in different clinical conditions [18–20]. In this study, we retrospectively collected clinical data of critically ill patients and selected LR, RF, GB, and SVC to predict NOAF risk, then compared the efficiency of these models to develop an optimized clinical prediction model for NOAF in critically ill patients.

## Materials and methods

### Study design and population

As a retrospective case-control study, our study followed the essentials of the STROBE guideline [21]. This study comprised a single-center, adult (≥18 years), general (non-cardiac surgical) ICU containing 23 beds. Electronic data of critical patients admitted to the ICU of the Affiliated Hospital of Nanjing University of Chinese Medicine from 1 August 2019–31 January 2022 were retrospectively analyzed at 8 February 2023. Patients who required intensive care or organ support therapy were included, regardless of the primary disease. All patients underwent continuous 5‑lead electrocardiogram monitoring for at least 48 hours. Patients with a history of paroxysmal or persistent AF before hospitalization, implantation of a permanent pacemaker, or pre-admission anti-arrhythmic drug therapy were excluded. Considering that retrospective studies inevitably have missing data, if the proportion of missing data is substantial, imputation methods may not yield robust results, so patients with more than 10% missing data were excluded to ensure data quality and the reliability of model analysis. NOAF was defined as the first documented episode of atrial fibrillation during ICU stay, characterized by the absence of P waves and irregular RR intervals, lasting for at least 30 seconds, and confirmed by continuous 5-lead ECG monitoring (Philips IntelliVue system) [9,22], which was extracted according to hospital diagnosis, related procedure or medical records, such as initiation of antiarrhythmic drugs or rate control therapy. The diagnosis of NOAF was confirmed by two independent senior ICU physicians.

### Ethical approval

This study was carried out in accordance with the Declaration of Helsinki, and the study protocol was approved by the Ethics Committee of the Affiliated Hospital of Nanjing University of Chinese Medicine (approval number: 2022NL-009–01).

### Consent statement

Considering that this was a retrospective clinical study, the data was analyzed anonymously, and authors could not identify individual participants during or after data collection. There was no direct involvement or impact on the individuals involved, and the research did not pose any risks or potential harm to the subjects, so the patient’s consent for participating in our study was waived by the Ethics Committee of the Affiliated Hospital of Nanjing University of Chinese Medicine.

### Data and information collection

General information obtained from patients’ medical records included age, sex, mean arterial pressure (MAP), oxygenation index (OI), heart rate (HR), history of chronic diseases such as coronary heart disease (CHD), heart failure (HF), diabetes, hypertension, chronic obstructive pulmonary disease (COPD), and chronic kidney disease (CKD). We also obtained laboratory and physiological data at admission to calculate the scores of sequential organ failure assessment (SOFA) and acute physiology and chronic health evaluation II (APACHE II). Other laboratory data, excluding the APACHE II and SOFA scores, such as procalcitonin (PCT), High-sensitivity C-reactive protein (Hs-CRP), Activated Partial Thromboplastin Time (APTT), D-dimer (DD), aspartate amino transferase (AST), MB isoenzyme of creatine kinase (CK-MB) and brain natriuretic peptide (BNP) were also collected. The data obtained were randomly classified into training and validation groups in a ratio of 8:2.

### Sample size calculation

Based on the literature, a presumed NOAF incidence of 20% was used to calculate the sample size of this study [5]. The sample size was calculated according to formula n =(1.96/δ)^2^φ (1 − φ) [23], where φ is the expected incidence of NOAF (φ = 0.20) and δ is the set margin of error (δ = 0.05), so the minimum sample size used for model construction was 246.

### Statistical analysis

Continuous variables were presented as median and interquartile range [25th percentile −75th percentile] or mean and standard deviation and were compared using *Mann-Whitney U test* or Student’s unpaired *t-test* depending on data distribution. Categorical data were presented as numbers and percentages and were compared using *Fisher’s exact probability method or chi-square test*. The clinical data and related laboratory results of the training and validation groups were compared. In the training cohort, potential predictors of NOAF were screened using two complementary machine learning methods: LASSO regression and Random Forest variable importance ranking. The intersection of both methods yielded a set of common predictive variables of NOAF. Receiver operating characteristic (ROC) curves of the confirmed predictors were subsequently plotted. Logistic Regression (LR), RF, Gradient Boosting (GB) and Support Vector Machine (SVC) models were utilized to ensure consistency, and each model contained the same input variables. The area under the ROC (AUROC) and precision-recall curve (AUPRC) were also used to evaluate the recognition ability of the different machine learning algorithms. Fivefold cross-validation, repeated 100 times for the four models, was plotted within the training set, utilizing AUROC as the optimization metric. In the validation set, confusion matrix and calibration curve were used to assess the degree of accuracy of the four models. After the above assessment, decision curve analysis (DCA) was conducted to evaluate the utility of the models in decision-making. The net reclassification index (NRI) and integrated discrimination improvement (IDI) were also calculated to evaluate the performance of the four models (calculated relative to LR as the baseline model). Learning curve was plotted to verify the reliability of different models. The SHapley Additive exPlanations (SHAP) was used to explain the supreme-performing model. To optimize model performance, we applied a grid search strategy using the GridSearchCV function with 5-fold cross-validation repeated 100 times. The optimization metric was AUROC. To address the class imbalance in the outcome variable, SMOTE (Synthetic Minority Oversampling Technique) was implemented within an imbalanced-learn pipeline. Each pipeline also incorporated standardization (StandardScaler) prior to model fitting to ensure consistent feature scaling. Statistical significance was defined as a two-sided *P*-value ≤0.05, and all data were analyzed using R software, version 4.2.3. Machine learning model construction, hyperparameter tuning, and performance evaluation were implemented in Python (version 3.11.3) using the scikit-learn and imbalanced-learn libraries.

## Results

### Patient characteristics

A total of 417 patients were included in this study, 102 patients among them had NOAF. The mean age of the patient was 72.51 ± 14.82 years and 284 (68.11%) patients were male. 333 patients were divided into the training set and 84 to the validation set, respectively. The distributions of baseline characteristics were similar between the two groups, and the incidence rates of NOAF were 81 (24.32%) and 21 (25.00%), respectively (Table 1).

**Table 1:** Baseline characteristics of patients in training and validation groups.

Characteristics	Total(*n* = 417)	Validation (*n* = 84)	Training (*n* = 333)	*P* value
Gender, *n* (%)	284 (68.106)	60 (71.429)	224 (67.267)	0.465^1^
Age, Mean±SD	72.513 ± 14.822	71.881 ± 14.127	72.673 ± 15.009	0.662^2^
Hypertension, *n* (%)	265 (63.549)	47 (55.952)	218 (65.465)	0.105^1^
CAD, *n* (%)	123 (29.496)	20 (23.810)	103 (30.931)	0.201^1^
DM, *n* (%)	162 (38.849)	38 (45.238)	124 (37.237)	0.179^1^
HF, n (%)	341 (81.775)	65 (77.381)	276 (82.883)	0.243^1^
CKD, *n* (%)	90 (21.583)	13 (15.476)	77 (23.123)	0.128^1^
COPD, *n* (%)	51 (12.230)	10 (11.905)	41 (12.312)	0.919^1^
APACHE, M (Q_1_, Q_3_)	21.000 (15.000, 25.000)	19.500 (14.000, 24.250)	21.000 (15.000, 25.000)	0.349^3^
SOFA, M (Q_1_, Q_3_)	8.000 (6.000, 10.000)	8.000 (5.000, 10.000)	8.000 (6.000, 11.000)	0.404^3^
MAP, Mean±SD	86.973 ± 19.098	87.465 ± 18.839	86.849 ± 19.189	0.792^2^
HR, Mean±SD	99.823 ± 25.132	99.226 ± 24.232	99.973 ± 25.387	0.808^2^
OI, M (Q_1_, Q_3_)	238.250 (163.600, 345.000)	231.710 (165.588, 340.250)	240.200 (163.600, 348.000)	0.788^3^
WBC, M (Q_1_, Q_3_)	10.040 (6.810, 14.900)	10.495 (6.702, 15.003)	9.850 (6.840, 14.900)	0.639^3^
hsCRP, M (Q_1_, Q_3_)	62.000 (17.660, 144.550)	50.405 (12.518, 144.822)	64.530 (18.140, 144.550)	0.471^3^
PCT, M (Q_1_, Q_3_)	1.050 (0.210, 6.490)	0.895 (0.203, 3.820)	1.100 (0.220, 7.420)	0.265^3^
DD, M (Q_1_, Q_3_)	2.980 (1.810, 6.730)	2.730 (1.600, 5.263)	3.030 (1.830, 6.930)	0.135^3^
APTT, M (Q_1_, Q_3_)	41.600 (37.000, 48.900)	42.300 (37.000, 49.700)	41.500 (36.900, 48.700)	0.881^3^
PT, M (Q_1_, Q_3_)	15.700 (14.400, 17.500)	15.400 (14.300, 17.125)	15.800 (14.500, 17.600)	0.247^3^
AST, M (Q_1_, Q_3_)	32.000 (19.000, 61.000)	33.500 (22.750, 65.500)	31.000 (18.000, 61.000)	0.435^3^
ALT, M (Q_1_, Q_3_)	33.000 (20.000, 69.000)	33.000 (19.750, 67.250)	33.000 (20.000, 70.000)	0.850^3^
TBil, M (Q_1_, Q_3_)	12.060 (7.500, 23.590)	10.675 (7.310, 23.910)	12.270 (7.530, 23.250)	0.570^3^
Scr, M (Q_1_, Q_3_)	99.500 (65.100, 227.300)	89.050 (57.325, 206.675)	103.300 (66.900, 234.100)	0.140^3^
CKMB, M (Q_1_, Q_3_)	13.000 (7.000, 23.000)	13.500 (7.000, 23.250)	12.000 (7.000, 23.000)	0.610^3^
BNP, M (Q_1_, Q_3_)	285.000 (128.000, 826.000)	246.000 (107.000, 742.000)	291.000 (134.000, 843.000)	0.355^3^
NOAF, *n* (%)	102 (24.460)	21 (25.000)	81 (24.324)	0.898^1^

^1^ *P* value between groups were assessed by *chi-square* test.

^2^ *P* value between groups were assessed by student’s unpaired *t-*test.

^3^ *P* value between groups were assessed by *Mann-Whitney U* test.

### Feature selection

As shown in S1 Fig, all Pearson correlation coefficients were less than 0.8, and all variables were included in the subsequent feature screening. LASSO regression and Random Forest variable importance ranking were first applied to perform feature screening, and then the intersection of both methods was identified as predictive variables. Five features were revealed as common predictors of NOAF: age, HR, MAP, APTT and BNP (S2 Fig). The ROCs of the five predictive variables were plotted and the AUROCs were 0.719, 0.598, 0.573, 0.556 and 0.636, respectively (S3 Fig). These variables were included in four machine learning algorithms (LR, RF, GB and SVC) to search for optimal hyperparameters and to construct different models to optimize the prediction model for NOAF.

### Construction and validation of clinical prediction models

Five selected predictors and optimized hyperparameters (LR: C = 1.0, penalty = ‘l2’, solver = ‘liblinear’; RF: n_estimators = 200, max_depth = 10, max_features = ‘sqrt’, min_samples_split = 2; GB: learning_rate = 0.1, n_estimators = 200, max_depth = 3, subsample = 1.0; SVC: lassifier – kernel = ‘rbf’, C = 1.0, gamma = ‘scale’, probability = True) were integrated into the clinical prediction model for NOAF. ROC and PRC were used to evaluate the recognition ability of the different models, and the AUROCs for LR, RF, GB and SVC in predicting NOAF were 0.762, 0.758, 0.728 and 0.751, respectively. The AUPRCs for the four models were 0.664, 0.524, 0.509 and 0.670, respectively (S1 Table). The calibration curves of the four models showed good consistency between actual results and predicted outcomes. While LR achieved the highest AUROC and SVC obtained the highest AUPRC, the RF model yielded the highest precision (0.464) and recall (0.650, tied with GB), along with the lowest Brier Score (0.176) and Log Loss (0.518), indicating superior calibration and balanced discrimination–calibration trade-off (S1 Table). Repeated fivefold cross-validation (100 iterations) further confirmed that RF maintained high and stable performance across multiple metrics, suggesting better generalization potential compared with the other algorithms (S4 Fig).

**Fig 1:**
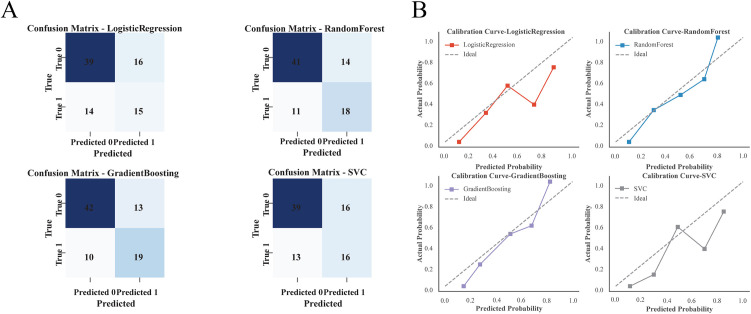
Confusion matrix and calibration curves of four models in validation cohort. A. Confusion matrix plots of four models. B. Calibration curves of four models.

**Fig 2:**
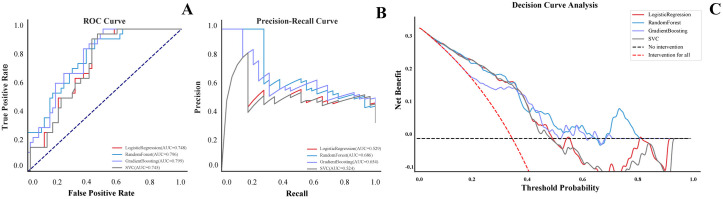
Receiver operator characteristic curve, Precision-Recall curve and decision curve analysis of four models in validation cohort. A. Receiver operator characteristic curves of four models in validation cohort. B. Precision-Recall curves in validation cohort. C. Decision curve analysis of four models in validation cohort.

In the validation group, the confusion matrices of LR, RF, GB and SVC were plotted, and the F1 scores of the four models were 0.500, 0.590, 0.623 and 0.525, respectively (Fig 1A, S2 Table). Calibration analysis revealed that the LR model demonstrated poor calibration, with a *Hosmer–Lemeshow test* p-value of 0.017, while the p-values for RF, GB, and SVC were 0.051, 0.284, and 0.053, respectively (Fig 1B). As *Hosmer–Lemeshow test* is not strictly applicable to non-parametric models (RF, GB, SVC), the other continuous metrics should be used to evaluate agreement between predicted probabilities and observed outcomes. Among the four models, the RF algorithm achieved the lowest Brier Score (0.132) and Log Loss (0.422), indicating the best overall calibration performance (S2 Table). ROC and PRC were also used to evaluate the recognition ability of the different models, and the AUROCs for LR, RF, GB and SVC were 0.748, 0.796, 0.799 and 0.745, respectively. The AUPRCs were 0.529, 0.686, 0.654 and 0.524, respectively (S2 Table, Fig 2A, 2B). The DCA of the four models revealed that when the threshold probability was between 10% and 60%, the net benefit of the RF algorithm was superior to those of the other three models (Fig 2C).

**Fig 3:**
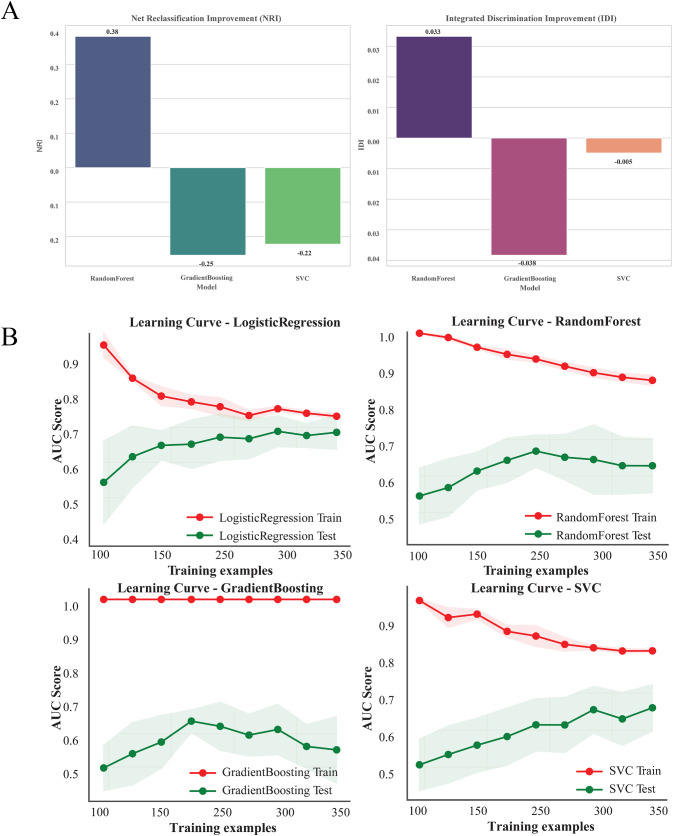
The net reclassification index and integrated discrimination improvement analysis and Learning curves of four models. A. The net reclassification index and integrated discrimination improvement analysis of four models (These metrics were calculated based on the Logistic regression model as the baseline). B. Learning curves of four models in validation cohort.

NRI and IDI were calculated to evaluate the performance of the RF, GB and SVC models relative to the baseline LR model. The NRI results showed that the RF significantly improved reclassification ability compared to the baseline model (NRI = 0.38). In contrast, GB (NRI = −0.25) and SVC (NRI = −0.22) performed slightly worse than the baseline in reclassification. The IDI results further demonstrated a moderate improvement in discrimination ability for the RF (IDI = 0.033) compared to the baseline, while GB (IDI = −0.038) and SVC (IDI = −0.005) showed no significant improvements (Fig 3A).

Considering the slight overfitting of the models in the validation cohort, the learning curve was used to verify the reliability of the different models and explore the reasons for overfitting. The results showed that the models showed satisfactory fitting except for the GB model, and RF also showed superior performance (Fig 3B). Even the RF model consistently achieved higher AUC scores in the validation set compared to the training set [AUROC (0.796 vs.0.758), AUPRC (0.686 vs.0.524)], suggesting that the model might have captured some details specific to the training data that may not generalize well to external datasets. However, as the size of the training set increased, the performance gap between the training and validation sets narrowed.

### Model explainability

**Fig 4:**
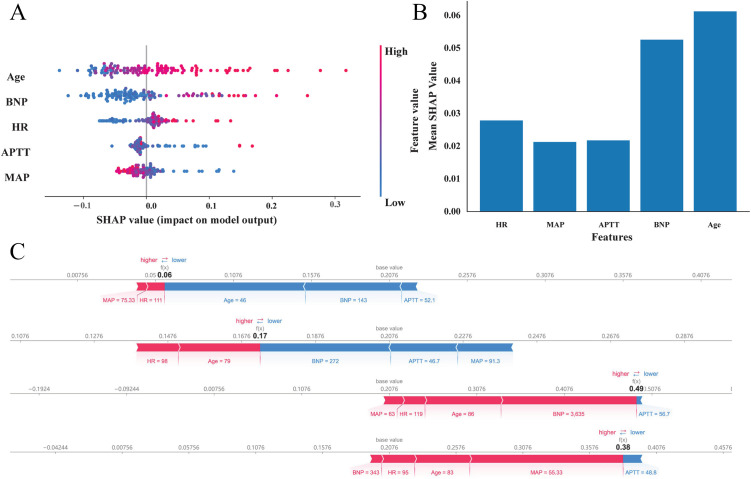
SHAP-based interpretation for the Random Forest model. A. The Beeswarm plot of five features across all model samples. B. Features ranked according to the mean absolute Shapley values. C. Local force plots of four cases (cases 1&2 were non-NOAF patients; cases 3&4 were NOAF patients).

The SHAP summary plot (Fig 4A, 4B) and local force plots of the four cases (Fig 4C) for the RF model were plotted. The Beeswarm plot showed that increased age, HR, APTT and BNP would elevate the risk of NOAF, while an elevated MAP indicated a diminished NOAF risk in ICU patients. According to the mean absolute Shapley values, age was the most significant risk factor for NOAF. Two non-NOAF patients (cases 1 and 2) and two NOAF patients (cases3 and 4) were illustrated in RF model explainer, and variables with red bars favored the occurrence of NOAF, and those with blue bars contradicted the result.

### Sensitivity analysis of optimized model

In consideration of age was an important predictor of NOAF, so additional DCAs were used to compare the expected net benefit of using age only and the full model integrated five predictors. The results demonstrated that while age alone provides moderate predictive performance, its clinical utility rapidly diminishes at higher threshold probabilities. In contrast, the full model demonstrated consistently higher net benefit across a clinically relevant threshold range (10%−60%) (S5 Fig).

## Discussion

### Principal findings

NOAF is one of the most common arrhythmias in the ICU, which could increase the risk of embolism and sudden death, and is also an indicator of poor prognosis [4,5]. Consequently, identifying modifiable risk factors, individualized assessment of patient-related risks, and targeted interventions are particularly vital for patient treatment.

In this study, we used LASSO regression and the RF algorithm to detect predictors of NOAF and found five predictors: age, HR, MAP, BNP and APTT. We then use four machine-learning algorithms (LR, RF, GB, and SVC) to develop and validate an optimized clinical prediction model for NOAF in critically ill adult patients. The AUROCs and AUPRCs of the four distinct machine learning algorithms showed that the RF model demonstrated superior performance with a good capacity for calibration and recognition. Although the AUPRC values of the RF model were 0.524 in the training cohort and 0.686 in the validation cohort, which may be interpreted as moderate in absolute terms, but considering approximately 24% event of NOAF in our population, and in such imbalanced datasets, AUPRC values are typically lower than AUROC values. What’s more, in many clinical prediction tasks, AUPRC values in the range of 0.6–0.7 have been shown to provide meaningful discrimination, particularly when accompanied by strong calibration and decision-analytic performance. In our analysis, the RF model achieved the highest AUPRC in the validation cohort, maintained stability across 100 repeated cross-validations. In addition, the RF model provides a better net benefit than the other three models. Furthermore, compared with the baseline LR model, RF achieved notable improvements in patient risk reclassification and discrimination. These findings suggest that, despite numerically moderate AUPRC values, the RF model provides clinically valuable and decision-supportive predictions for NOAF in critically ill patients. Although the RF model demonstrated stable performance in both the training and validation sets, the learning curves indicated slight overfitting, but increasing sample size may further improve the model’s generalizability.

The SHAP summary plot of the RF model showed that age was the most important predictor of NOAF in critically ill patients, which is consistent with previous studies. Studies have indicated that AF morbidity generally increases with age. It occurs in less than 1% of people aged between 60 and 65 years and in more than 10% of people older than 80 years. Moreover, the prevalence is higher in men and seems higher in White persons than Black persons [9]. Studies also revealed that ageing was an independent risk factor associated with NOAF both in the Asian and Western populations [24]. Our study found that age was the primary predictor of NOAF (OR=1.07, 95%CI 1.04–1.10, P < 0.001) and AUROC of age for prediction of NOAF was 0.719. The DCAs demonstrated that while age alone provides moderate predictive performance, its clinical utility rapidly diminishes at higher threshold probabilities. In contrast, the full model achieved an AUROC of 0.796 and demonstrated consistently higher net benefit across a clinically relevant threshold range (10%−60%).

BNP is a biomarker released from cardiomyocytes and is stimulated mainly by myocardial stretching. Higher levels of BNP and NT-pro BNP were risk predictors for AF regardless of the size of the left atrial or left ventricular function [25]. The relationship indicates that a higher level of BNP corresponds to a higher risk of AF occurrence and relapse. Stretch-induced sarcoplasmic reticulum calcium leak could increase susceptibility to AF, and concomitant increased BNP secretion would be relevant to arterial stiffness, which should contribute to the development of AF [26,27]. NOAF in ICU patients may have different risk factors from AF seen in the general population, but there may be common mechanisms, such as electrical and structural remodeling of atriums [28]. Studies found that neurohumoral changes, such as BNP, may contribute to AF-related atrial structural remodeling, and a high plasma BNP level was an independent risk factor for NOAF [29]. Our study showed similar results and increased admission BNP was linked to a higher risk of NOAF. Existing research has shown that therapeutic strategies based on the molecular mechanisms of natriuretic peptide (NPs) regulation and function may improve the outcomes of cardiovascular diseases, which may provide a potential measure for NOAF prevention in critically ill patients [30].

The association between HR and cardiovascular and/or all-cause mortality is an intriguing research topic in the cardiovascular department and in critical care medicine. Studies have revealed that increased HR is an independent predictor of cardiovascular disease, which can lead to increased rates of arrhythmia and cardiac dysfunction [31]. Increased HR is also a marker of hemodynamic instability, deterioration of organ function, and poor prognosis in critically ill patients [32]. A dose-response meta-analysis also revealed that high resting HR was associated with an increased risk of AF [33]. We found that higher HR at admission could increase the probability of NOAF in ICU patients. Studies have revealed that beta-blockers could be used as first-line therapy for HR control, and β-blocker treatment in ICU patients was associated with better HR control and reduced mortality [3,34,35]. These results suggest that reducing resting HR may decrease the risk of NOAF and improve the outcome of critical patients.

Other considerable predictors in our prediction model included APTT and MAP. APTT is a common and fundamental test to screen for coagulation disturbances and is used to monitor anticoagulant therapy, assess clotting factor deficiencies, and evaluate treatment effects. Our study found that prolonged APTT may be accompanied by an increased risk of NOAF (OR 1.02, 95%CI 1.01–1.03, P = 0.002). Recent studies found that isolated prolongation of APTT was not just bleeding risk and a common conditions of ICU patients [36]. Prolonged APTT may serve as surrogates for other underlying factors, such as systemic inflammation, coagulopathy (such as activation of the fibrinolytic system) and structural or functional changes in the atria (such as left atrial enlargement or fibrosis). These factors may collectively contribute to the development of AF. Additionally, changes in APTT could be influenced by confounding factors, such as elevated inflammatory status or imbalances in the coagulation-fibrinolysis system, which may indirectly increase the risk of NOAF. Based on these, it was not the prolonged APTT that “caused” NOAF, but results affected by other unknown factors, and further research is needed to clarify the real correlation between APTT and NOAF.

Many studies focusing on the risk of AF have shown that arterial stiffness and hypertension are independent predictors [37]. A meta-analysis about intraoperative hypotension and postoperative outcomes has shown that a target intraoperative mean arterial pressure ≤60 mm Hg cannot increase mortality can but can reduce rate of atrial fibrillation [38]. Studies have found that catecholamine vasopressor treatment is associated with an increased risk of NOAF [39,40]. Our study found that the proportions of hypertension between the NOAF and non-NOAF groups were similar, but MAP at admission was slightly higher in the non-NOAF group, nevertheless the differences were not significant (84 ± 20 mmHg vs. 88 ± 19 mmHg, P = 0.06). A possible explanation for these conflicting results is that a history of hypertension and concomitant artery stiffness are risk factors for AF; however, catecholamine vasopressors used to rescue hypotension or shock in ICU patients might also increase the risk of NOAF. Therefore, prospective studies are required to further understand the correlation between MAP and NOAF.

Based on the above mentioned five predictors, the RF model displayed satisfactory discrimination and calibration in both the training and validation sets, with AUROCs of 0.758 and 0.796, respectively. Additionally, the RF model showed better clinical utility with a higher net benefit in DCA analysis. These analyses revealed that the RF model has the potential to help intensivists identify high-risk patients and make treatment decisions.

### Innovations and limitations

#### Innovations of this study.

This study developed a NOAF prediction model based on dynamic physiological and laboratory indicators in ICU patients, with the RF model demonstrating the best performance (validation cohort ROC AUC = 0.796). Compared with existing AF prediction models, this study’s innovations include: (1) a unique study population focusing on critically ill patients rather than the general population; (2) the inclusion of dynamic physiological and laboratory variables, which are more relevant to acute pathophysiology; (3) the application of machine learning to optimize model performance; and (4) the use of SHAP analysis to identify the importance of key features.

#### Limitations of this study.

This study is a retrospective study and has certain limitations. First, the single-center design and relatively small sample size, particularly in the validation cohort, restrict the ability to apply these results universally across diverse healthcare settings or populations and the unique characteristics of the patient cohort and local practices may not reflect broader trends or conditions encountered in other hospitals or regions. The learning curves indicated slight overfitting, which is likely attributable to the limited data volume. Although repeated cross-validation and internal holdout validation demonstrated internal stability, these techniques cannot substitute for true external validation. Future studies should incorporate multicenter datasets to evaluate the model’s generalizability across diverse ICU settings and populations. Expanding the dataset will also help improve the model’s robustness and reduce potential overfitting; second, echocardiographic parameters such as atrial size and left ventricular function are well-established predictors of atrial fibrillation, while some patients failed to gain a comprehensive echocardiographic examination to assess the structure and function of the heart, which prevented us from analyzing the relationship between atrial structure and occurrence of NOAF, which is a notable limitation. Nevertheless, this limitation also reflects a practical advantage for real-world application: echocardiographic data is not always available or timely, especially in patients requiring urgent decision-making. By relying solely on routinely collected clinical and laboratory variables, our model remains feasible and generalizable across diverse ICU settings, including resource-limited environments. Further studies should explore these echocardiographic parameters at admission and assess their effect on the model’s performance; third, only occurrence of NOAF was observed, and the effects of treatment strategies, such as mechanical ventilation and fluid resuscitation, on the occurrence of NOAF were not evaluated. Additionally, the new occurrence of AF after admission to the ICU does not exclude patients who have similar asymptomatic episodes in the past, these patients were not real NOAF; finally, retrospective study relies on existing data, making it challenging to establish causal relationships between predictors and outcomes. This limitation restricts the study of hypothesis generation rather than definitive conclusions, so further prospective validation research is needed to evaluate the exact relations between NOAF and these risk factors.

## Conclusions

In conclusion, we used machine learning techniques to build a prediction model for NOAF in critical patients, and the validated RF model had the best performance, showing good calibration and discrimination, as well as superior clinical utility compared to the LR, GB and SVC models. The screened predictors, such as age, HR, MAP, BNP and APTT, could assist in making clinical decisions and predicting the prognosis of ICU patients. From a clinical perspective, the proposed RF-based prediction model, which relies only on routinely available clinical and laboratory variables, can automatically generate risk scores without requiring additional diagnostic testing or manual data entry. It has the potential to be integrated into ICU electronic medical record (EMR) systems for real-time risk assessment of NOAF. In resource-limited settings, the model’s simplicity and low data dependency further support its broad applicability. For further studies, we should evaluate its real-time clinical performance and try to integrate other potential risk factors to improve the model’s performance and extend its application to different patients and settings.

## Supporting information

S1 Table Performance metrics for prediction models in the training cohort. (DOCX)

S2 Table Performance metrics for prediction models in the validation cohort. (DOCX)

S1 Fig Pearson correlation matrix of clinical variables. (TIF)

S2 Fig Predictive variables screened by LASSO regression and Random Forest algorithm. A. Variable selection by using LASSO regression algorithm. B. Variable selection by using Random Forest algorithm. C. Five variables were determined by LASSO regression and Random Forest algorithm.
(TIF)

S3 Fig Receiver operator characteristic (ROC) curves of five independent predictive variables for predicting NOAF. (TIF)

S4 Fig Metrics of four models with fivefold cross-validation repeated 100 times in training cohort. (TIF)

S5 Fig Decision curve analysis of age and the full model. (TIF)

S1 File The data points of 417 patients enrolled in the study. (PDF)
