# Supplementary material for: Optimizing clinical prediction model for new-onset atrial fibrillation in critically ill patient: Based on machine learning
Source: PLoS One. 2025 Sep 11;20(9):e0331857. doi: 10.1371/journal.pone.0331857 (PMC12425216; doi:10.1371/journal.pone.0331857)
Supplement: S1 Table — (DOCX) [file pone.0331857.s001.docx]

S1 Table: Performance metrics for prediction models in the training cohort.

| Model | Accuracy | Precision | Recall | F1 score | ROC AUC | PR AUC | Brier score | Log Losse |
| --- | --- | --- | --- | --- | --- | --- | --- | --- |
| Logistic Regression | 0.639 | 0.353 | 0.60 | 0.444 | 0.762 | 0.664 | 0.199 | 0.572 |
| Random Forest | 0.735 | 0.464 | 0.65 | 0.491 | 0.758 | 0.524 | 0.176 | 0.518 |
| Gradient Boosting | 0.711 | 0.433 | 0.65 | 0.520 | 0.728 | 0.509 | 0.195 | 0.575 |
| Support Vector Machine | 0.602 | 0.324 | 0.60 | 0.421 | 0.751 | 0.670 | 0.203 | 0.585 |
