# Supplementary material for: Optimizing clinical prediction model for new-onset atrial fibrillation in critically ill patient: Based on machine learning
Source: PLoS One. 2025 Sep 11;20(9):e0331857. doi: 10.1371/journal.pone.0331857 (PMC12425216; doi:10.1371/journal.pone.0331857)
Supplement: S2 Table — (DOCX) [file pone.0331857.s002.docx]

S2 Table：Performance metrics for prediction models in the validation cohort.

| Model | Accuracy | Precision | Recall | F1 Score | ROC AUC | PR AUC | Brier Score | Log Loss |  |  |
| --- | --- | --- | --- | --- | --- | --- | --- | --- | --- | --- |
| Logistic Regression | 0.643 | 0.484 | 0.517 | 0.500 | 0.748 | 0.529 | 0.207 | 0.595 |  |  |
| Random Forest | 0.702 | 0.562 | 0.621 | 0.590 | 0.796 | 0.686 | 0.132 | 0.422 |  |  |
| Gradient Boosting | 0.726 | 0.594 | 0.655 | 0.623 | 0.799 | 0.654 | 0.181 | 0.547 |  |  |
| Support Vector Machine | 0.655 | 0.500 | 0.552 | 0.525 | 0.745 | 0.524 | 0.209 | 0.600 |  |  |
