# Supplementary figures and images for: Optimizing clinical prediction model for new-onset atrial fibrillation in critically ill patient: Based on machine learning
Source: PLoS One. 2025 Sep 11;20(9):e0331857. doi: 10.1371/journal.pone.0331857 (PMC12425216; doi:10.1371/journal.pone.0331857)

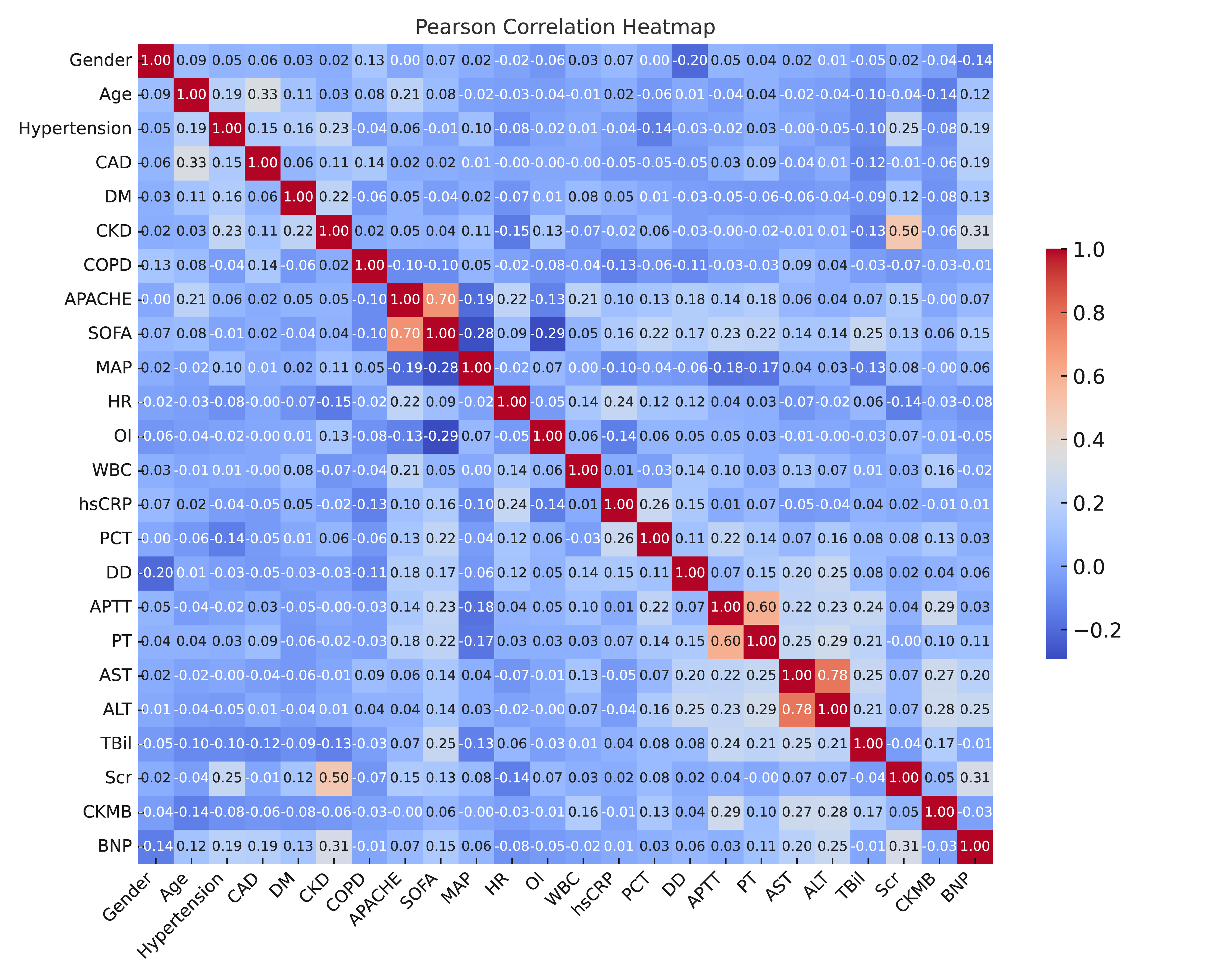

Supplement: S1 Fig — (TIF) [file pone.0331857.s003.tif]

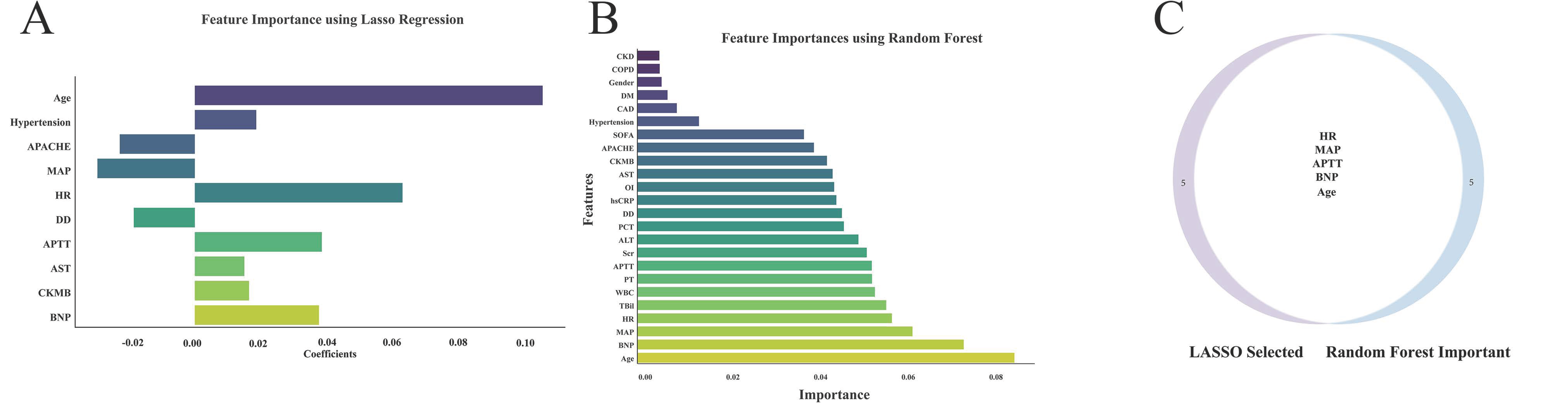

Supplement: S2 Fig — A. Variable selection by using LASSO regression algorithm. B. Variable selection by using Random Forest algorithm. C. Five variables were determined by LASSO regression and Random Forest algorithm. (TIF) [file pone.0331857.s004.tif]

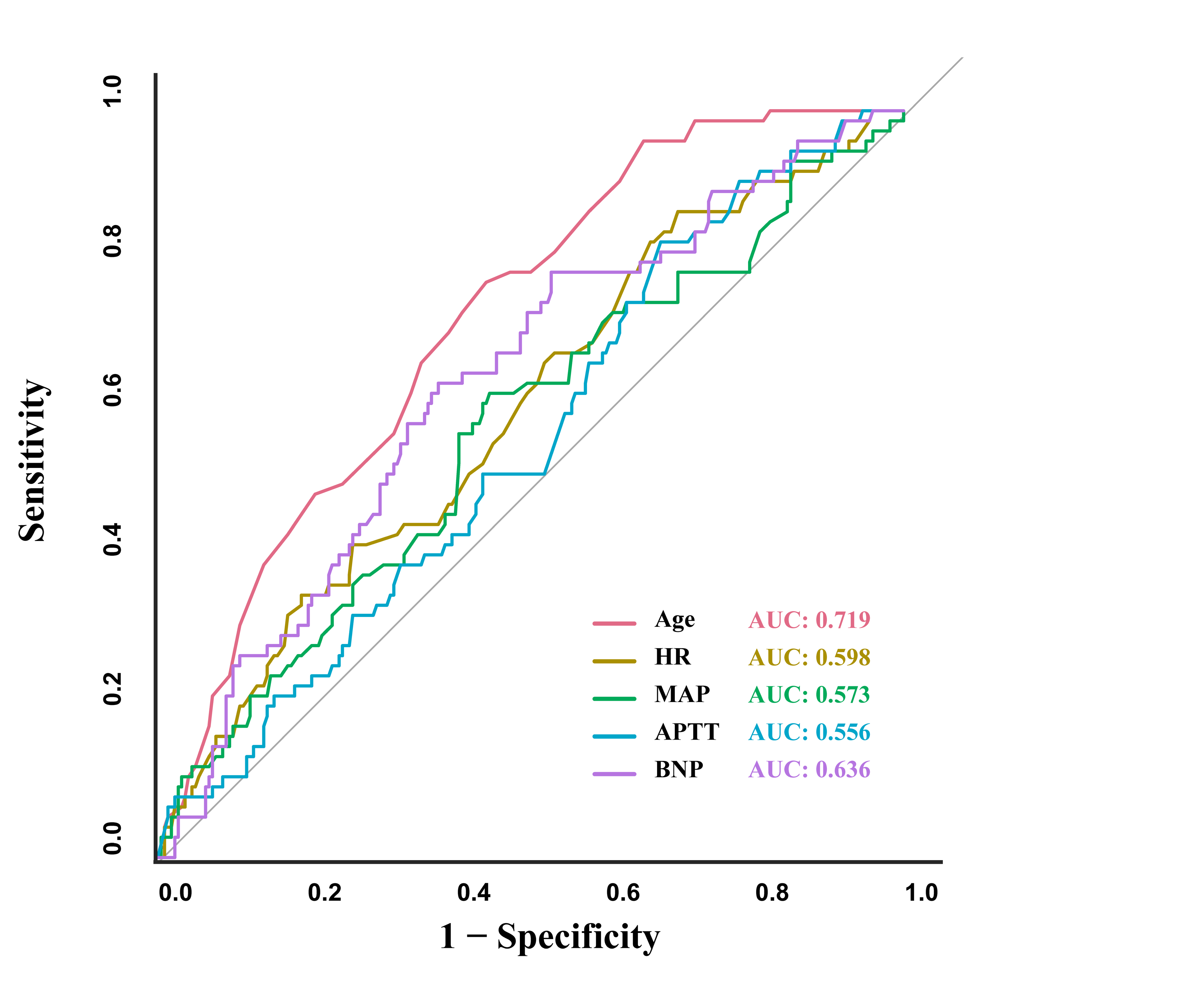

Supplement: S3 Fig — (TIF) [file pone.0331857.s005.tif]

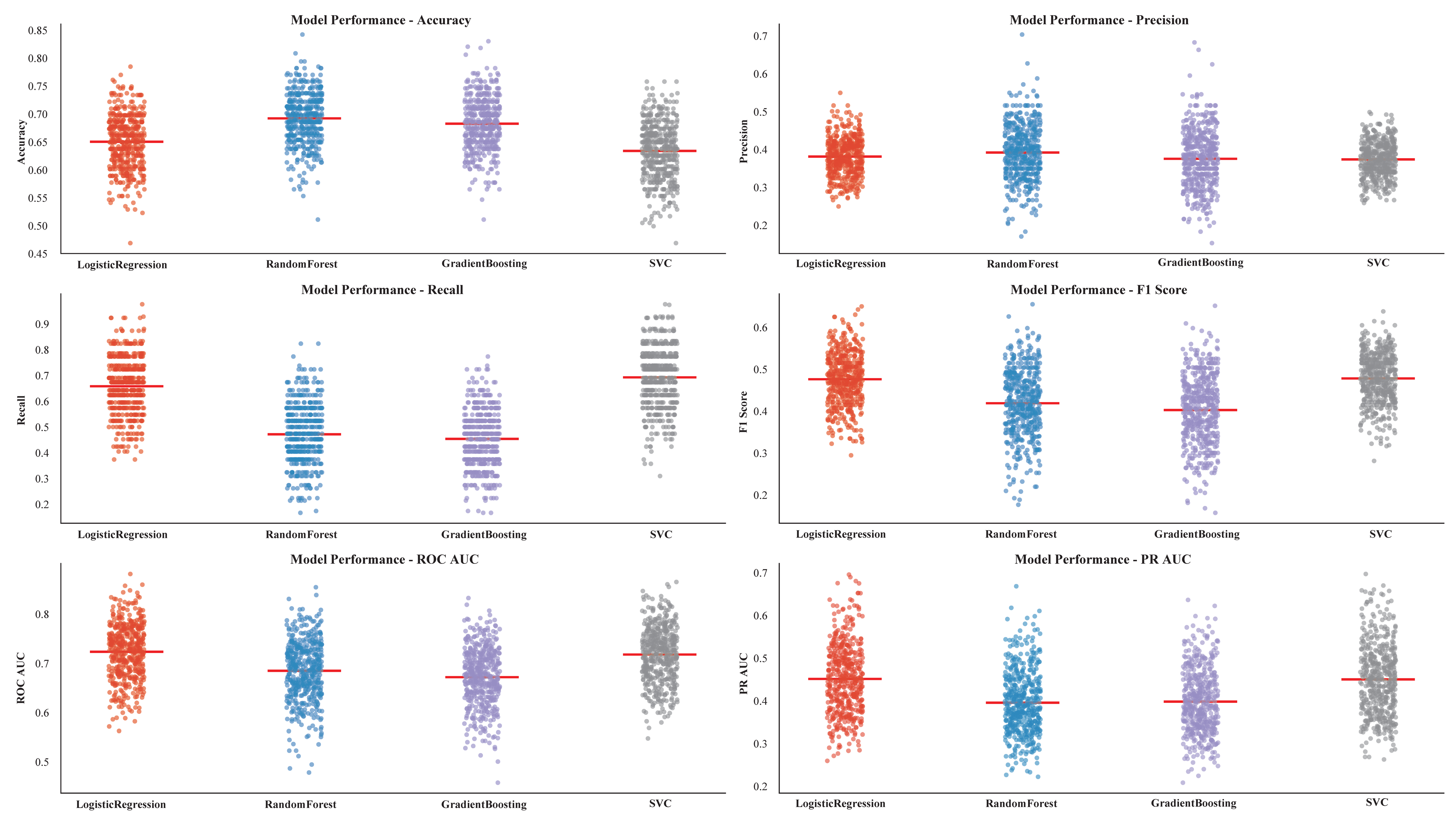

Supplement: S4 Fig — (TIF) [file pone.0331857.s006.tif]

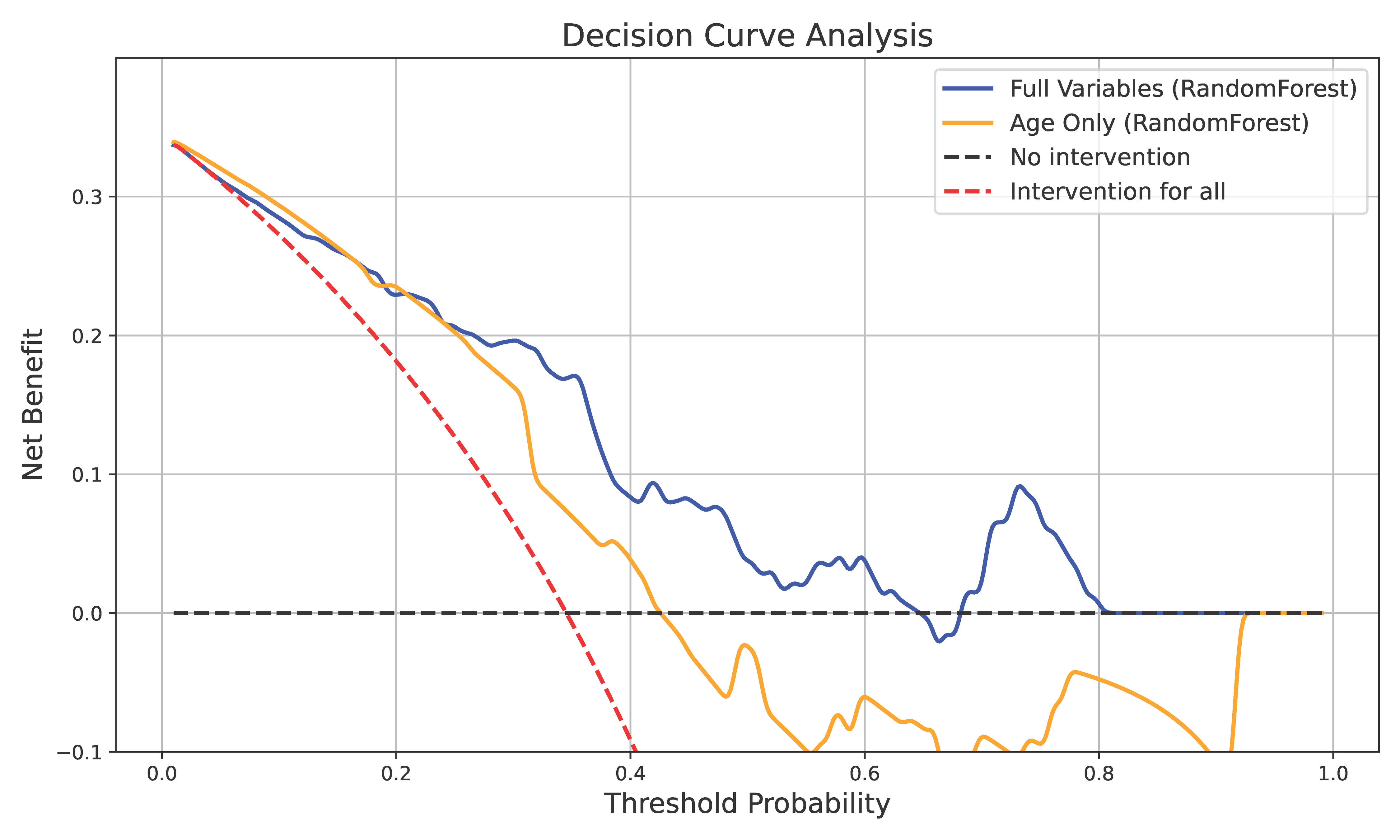

Supplement: S5 Fig — (TIF) [file pone.0331857.s007.tif]
