# Supplementary material for: Optimizing clinical prediction model for new-onset atrial fibrillation in critically ill patient: Based on machine learning
Source: PLoS One. 2025 Sep 11;20(9):e0331857. doi: 10.1371/journal.pone.0331857 (PMC12425216; doi:10.1371/journal.pone.0331857)
Supplement: S1 File — (PDF) [file pone.0331857.s008.pdf]

No.	gender	age	pertens	CAD	DM	CKD	COPD	APACHI	SOFA	MAP	HR	OI	WBC	hsCRP	PCT	DD	APTT	PT	AST	ALT	TBil	Scr	CKMB	BNP	NOAF
1	1	79	1	0	1	0	0	11	6	106	134	213.2	10.89	30	0.48	0.53	44.7	17.7	71	34	15.82	66.7	7	284	1
2	0	80	1	0	0	0	0	23	8	41.7	170	314.3	13.87	170	39.88	19.65	40.2	16.4	28	40	4.52	373.6	20	236	1
3	0	89	1	1	0	0	0	25	8	123	100	223.6	11.7	3	3.69	1.58	59.6	16.1	1075	1346	28.31	279.7	13	4711	1
4	1	63	1	0	0	0	0	6	2	97	107	170	11.75	240	0.1	20	57.1	18.2	98	115	16.38	66.9	412	36	1
5	0	78	1	1	1	1	1	23	6	50	90	277.6	20.72	8	0.74	3.04	48.5	14.4	16	46	3.98	562.1	29	826	1
6	1	68	1	0	0	0	0	15	10	71	125	105.8	3.66	195	0.65	2.55	41.4	12.7	28	64	13.72	54.7	22	29	1
7	1	88	1	0	0	1	0	42	16	53.6	113	91.7	8.2	147	4.21	5.57	75.8	21.6	55	73	10.24	311	14	128	1
8	1	80	1	1	0	1	1	31	13	103.7	178	136.6	6.8	42	0.31	0.79	36.9	13.8	61	84	10.54	150.1	39	560	1
9	1	69	1	0	0	0	0	39	16	53	150	117.2	41.67	76	50	8.87	177.5	48.6	167	356	104.9	259	28	577	1
10	0	89	0	0	0	0	0	13	3	84	90	375	5.6	7	0.1	0.27	43.7	13.1	15	17	3.95	59.4	4	25	1
11	0	88	0	0	1	0	0	24	7	82	87	244.2	12.36	9	1.05	20	43.7	27.7	1811	2612	55.5	230.2	12	2142	1
12	1	75	0	0	0	0	1	13	5	105	124	344	8.6	26	0.1	2.95	33	13.9	23	21	3.41	40.8	35	126	1
13	1	81	1	0	1	1	0	33	12	90.3	60	300	12.97	169	96	14.15	46.5	16.2	78	74	17.86	401.9	14	723	1
14	1	85	1	1	1	0	0	32	16	76	130	131.1	1.38	37	18.8	2.3	32.8	16.8	56	65	25.11	176.8	2	55	1
15	0	85	1	0	1	0	0	27	11	98	57	167	8.38	3	0.21	2.01	37.8	14.3	10	19	11.76	97	14	367	1
16	1	84	1	1	1	0	0	14	6	88	71	113.7	10.71	47	0.48	1.71	37.3	14.2	18	14	7.6	103.8	10	579	1
17	1	72	1	1	0	0	1	12	5	81	87	348.6	5	6	0.1	5.4	33.3	14.5	23	35	24.06	71.3	23	107	1
18	1	68	1	1	1	0	0	14	5	83	122	147	18.19	170	5.12	1.12	39.6	15	19	16	3.24	40.7	18	241	1
19	1	84	1	0	1	0	1	16	6	82	96	164.8	16.8	41	0.52	2.33	35.5	14.7	337	269	12.97	55.2	10	137	1
20	1	87	0	1	0	0	1	21	10	42.6	91	92.1	6.65	55.71	0.23	1.17	43.4	16.4	42	26	15.5	50.8	1	292	1
21	0	70	1	0	1	0	0	25	11	85	130	130	15.8	74	2.93	9.47	34.8	16.7	23	38	83.51	82	20	295	1
22	1	78	1	0	0	1	0	22	11	75.6	139	345	8.82	97	17.02	1.63	51	17	39	74	24.31	284	3	1167	1
23	0	84	1	1	0	0	0	21	14	108	98	160.2	7.06	12	2.92	8.02	49.8	20.1	14	49	30.74	125.5	16	1802	1
24	1	91	1	0	1	0	0	21	7	80	102	484	10.13	190	45.42	3.83	38.3	17.7	38	83	14.25	72.4	14	1019	1
25	0	92	1	0	1	0	0	13	3	93.3	100	547.8	6.02	24	0.58	3.93	43.5	14.2	30	67	10.65	78.7	27	1989	1
26	1	79	1	0	0	0	0	23	11	83.6	96	422.5	19.12	67	72.29	3.9	46.4	16.5	152	117	23.92	75.4	10	354	1
27	1	83	1	0	1	1	0	18	10	85.3	89	365.9	7.23	5	0.79	4.4	180	20	64	130	12.88	150.4	88	100	1
28	1	86	1	1	1	0	0	13	10	104.3	94	146.3	28.65	310	50	5.12	41.2	13.5	49	66	6.72	222.9	80	136	1
29	1	67	1	0	0	0	0	21	9	101	96	175	15.36	6	0.1	1.09	36.6	16.4	11	27	15.73	79	14	127	1
30	1	81	0	1	1	0	0	19	11	86	89	392	4.34	210	323	2.29	49.2	16.6	18	28	7.71	205	26	442	1
31	0	85	1	0	1	0	0	30	13	75.6	130	426.8	6.12	81	4.08	13.47	55.2	24.3	1445	849	50.68	340.2	21	3558	1
32	1	77	0	0	0	0	0	22	11	64.3	121	224	14.68	200	49.1	6.68	48.5	17.2	102	211	6.33	563	58	778	1
33	1	71	1	0	1	0	0	30	8	107.6	135	208.5	13.58	180	3.53	9.24	43.3	16.8	21	30	3.55	227.3	1.5	685	1
34	1	79	1	0	1	1	0	37	13	68.6	109	522	23	160	342.5	3.14	180	29.6	535	1193	23.25	546.2	3	1791	1
35	0	68	0	0	0	0	0	12	4	90.3	136	287.5	22.96	98	2.75	20	36.8	19.8	38	102	23.26	89	37	1734	1
36	1	77	0	0	0	0	0	8	8	74	74	394.6	2.42	5.16	1.92	1.45	42.5	12.3	112	585	53.51	190.2	68	400	1
37	1	85	0	1	1	1	0	13	5	73.6	76	216	5.57	4	0.11	0.88	39.2	14.1	12	15	3.11	58.6	13	269	1
38	1	89	0	1	0	0	0	23	10	129	110	267.5	16.79	41	0.56	1.89	41.3	15.2	33	85	9.27	58.7	7	1039	1
39	1	95	1	1	0	0	0	21	6	98	104	419.8	3.59	190	13.29	1.51	180	120	16	47	25.93	97.4	16	1598	1
40	0	75	1	1	0	0	1	7	2	102.6	95	284	15.43	0.5	0.1	1.83	34.5	14.1	29	41	4.49	58.7	39	1870	1
41	0	89	0	0	0	0	0	14	4	75	94	365.9	6.41	49.76	0.37	5.49	36.6	12.6	11	27	13.6	50.4	9	843	1
42	1	72	1	0	0	0	0	29	13	56	137	112	9.73	86.89	5.43	0.34	37.8	14.9	38	31	18.5	70.9	3	508	1
43	1	89	0	1	0	0	0	24	9	119.3	112	338	14.9	40	0.62	1.66	39.4	15.7	18	25	13.85	93.7	15	156	1
44	1	95	1	1	0	0	0	20	8	83	87	351.2	5.5	38.18	0.1	1.22	37.1	14.1	48	52	6.45	51.5	8	2169	1
45	1	74	1	0	0	0	0	22	12	91.6	83	177.3	14.42	68.73	0.36	6.76	39	13.7	16	26	32.64	80.2	14	137	1
46	0	76	1	0	1	0	0	26	9	73	95	339	12.24	7.94	1.2	7.24	40.1	23.1	494	930	8.54	45.3	22	606	1
47	1	71	0	0	0	0	0	9	2	91.6	87	260.2	6.66	121.3	0.56	2.98	31.6	14	65	94	7.97	69.7	11	86	1
48	1	86	1	1	0	1	0	10	5	77	77	436.1	11.13	137.2	0.64	7.36	45.3	15.9	37	39	10.6	288.6	3	1810	1
49	1	61	1	0	0	0	0	26	8	83.3	108		11.36	230	0.68	3.51	31.2	16.1	32	82	6.56	116.2	7	209	1
50	1	76	1	0	0	0	0	5	2	84.6	128	236.1	8.7	241.5	3.27	8.39	46.3	20.3	36	24	15.67	65.7	3	239	1

51	0	78	1	1	0	0	0	24	5	146.3	68	397.3	14.79	16.67	0.1	1.15	36.1	16.1	32	54	4.95	55	19	374	1
52	0	77	1	1	1	1	0	23	9	78.6	92	661.4	20.16	259.7	25.8	20	43.2	18.9	22	36	22.3	982.2	28	125	1
53	0	84	1	0	1	0	0	11	4	112.7	102	554	11.55	106.9	0.29	2.71	43.2	13.8	17	10	8.59	79	13	875	1
54	1	83	1	1	1	1	0	12	9	74.3	85	236.6	3.94	75.09	0.42	2.81	41.2	15.1	25	17	4.97	577.9	16	2473	1
55	0	85	1	1	0	1	0	18	6	106.7	146	147	3.27	23	0.17	7.65	34.6	14.4	27	32	7.53	60.7	23	658	1
56	1	79	1	1	0	0	1	12	7	91.3	98	256.8	11.12	151.8	2.09	1.64	46.7	15.3	27	18	12.05	89.2	21	272	1
57	0	89	1	0	0	0	0	34	14	66	132	137	25.63	107.3	2.11	9.23	37.2	16	13	32	41.76	73.1	9	1372	1
58	0	82	0	0	1	1	0	17	7	83.6	67	299	10.17	91.53	0.13	3.28	54.5	17	35	40	6.66	220.7	13	1078	1
59	1	86	1	1	0	0	0	21	9	90.3	98	170	10.51	103.8	0.82	2.65	57.9	17.5	7	43	5.92	220	3	444	1
60	1	53	1	0	0	0	0	17	6	73	130	101.3	3.34	81.67	0.27	1.6	37.6	15	72	78	7.32	45.6	10	29	1
61	0	61	0	0	0	0	0	27	12	82.7	111	178	8.36	66.89	176	2.89	180	38.6	1612	2457	51.25	234.1	653	124	1
62	1	62	1	1	0	0	1	12	7	90	132	230.2	8.86	0.5	0.01	6.95	180	16.2	59	99	31.66	114.6	149	31	1
63	1	65	0	0	0	0	1	11	4	92	89	130.7	15.62	16.66	0.05	1.41	32.8	15.2	6	33	22.8	81.5	34	78	1
64	0	88	1	1	0	0	0	22	11	54	63	190.8	6.81	16.92	0.25	7.37	48.5	15.8	20	42	60.85	71.3	96	1628	1
65	1	71	0	0	0	0	0	21	10	68.6	99	335.6	15.61	28.99	0.59	7.71	180	18.1	40	26	27.32	155.1	24	55	1
66	0	70	0	0	1	1	0	16	7	76.3	81	415	25.38	159.7	1.41	3.05	45.2	14.7	25	47	22.7	557.2	43	2366	1
67	1	75	1	0	1	1	0	8	8	107.6	97	163.6	3.09	1.8	0.01	1.94	40.8	15.6	43	21	10.42	427	9	1460	1
68	1	94	0	1	1	0	0	20	11	72	75	129.5	14.6	14.6	0.78	4.59	38.8	17.2	28	57	27.46	125.7	7	390	1
69	1	77	1	1	0	0	0	6	9	83	95	134	15.38	87.6	2.11	6.85	59.1	30.9	1678	2421	43.63	214.6	31	1593	1
70	0	89	1	1	0	0	0	23	7	83	164	431.2	8.55	6.34	6.31	2.81	38.1	16.2	15	20	29.45	77	10	883	1
71	1	75	1	0	0	1	0	17	7	107.6	89	137.2	17.65	133.6	5.15	3.03	38.8	17.8	78	92	4.56	1078	23	2472	1
72	0	57	1	1	0	0	0	12	5	68.3	90	442.5	9.72	1.25	0.25	2.45	41.4	15.4	35	40	9	62.1	34	2454	1
73	1	71	1	1	1	0	0	25	9	75	141	272.9	6.54	216.8	2.99	1.21	37.7	15.5	34	24		56.6	14	474	1
74	1	91	0	0	1	0	0	23	8	58.6	89	245	16.8	101.6	0.05	0.8	36.7	16.1	23	21	12.06	99.5	15	878	1
75	1	70	0	0	0	0	0	26	11	79	120	240.5	3.06	213.3	21.3	3.75	56	21.6	27	32	23.84	132.1	27	127	1
76	1	72	1	0	0	0	0	13	6	70	104	92.7	13.28	13.16	10.16	6.4	36.7	14	249	151	19.7	103.3	29	1323	1
77	0	90	1	1	0	0	0	24	5	134	95	470	12.15	96.69	6.96	12.52	34.3	15.7	61	19	23.5	103.6	36	547	1
78	1	83	0	0	0	0	0	18	6	104.6	102	112.7	12.82	265.8	1.65	5.23	41	15.3	72	95	62.8	95.9	12	321	1
79	0	66	1	0	1	1	0	30	8	75.3	110	411	10.07	296.8	27.58	3.72	35	15.4	12	11	16.1	548.2	12	927	1
80	1	65	0	0	1	0	0	24	11	83	126	190.8	3.56	64.34	2.59	13.22	38.9	14.3	186	57	10.84	184.9	26	76	1
81	1	83	1	0	0	0	0	37	14	70	121	56.67	9.24	326.1	52.44	2.7	50.1	14.8	159	37	26.5	202.6	44	2032	1
82	1	97	1	1	0	1	1	28	11	82.33	101	154.8	5.17	78.48	2.28	2.28	51	17.2	37	18	14.7	289.6	10	1731	1
83	1	70	1	1	1	1	0	22	10	98.33	102	528.5	24.46	146.4	>100	2.83	46.9	18.3	111	46	28.7	756.6	18	760	1
84	1	90	1	0	1	0	0	35	8	50.33	121	205.3	20.12	17.06	1.33	3.74	56.7	19.2	27	7	6.9	358.4	11	254	1
85	1	83	1	0	0	0	0	30	8	55.33	95	257.5	7.17	235	23.31	5.3	48.8	16.5	71	51	7	401.3	136	343	1
86	0	87	0	0	1	0	0	22	3	38.33	141	312	7.84	104.5	0.26	2.42	39.3	15.7	23	237	17.61	79.7	3	149	1
87	1	68	1	0	0	0	0	16	3	109	112	240.4	12.36	190.9	1.78	4.41	38	16.1	99	130	12.4	63.8	11	171	1
88	0	96	0	0	0	0	0	19	5	87.33	86	136	12.69	23.29	0.05	1.04	39.2	13.7	30	21	2.84	46.2	17	78	1
89	1	75	0	0	1	1	1	25	8	104.6	117	262.9	14.73	195.5	9.39	0.76	52.5	19.3	14	10	11.2	160.8	8	91	1
90	0	75	0	0	0	0	0	30	15	68.33	90	112.6	11.14	144.6	38.09	10.31	129.7	25.4	84	31	297.3	280.2	13	763	1
91	0	86	1	0	1	0	0	29	14	63	119	83.44	15.6	282	11.47	14.49	56.7	18.6	16	15	22.13	158.1	9	3635	1
92	1	81	1	0	0	0	0	27	15	66.33	136	93.5	8.57	283.7	26.78	3.35	47.4	15.6	19	11	26.96	63.8	9	1284	1
93	1	76	1	0	1	0	0	24	4	85	113	401	20.05	5.3	0.23	3.37	77.8	15.3	52	28	28.6	100.1	25	734	1
94	0	87	0	0	0	0	0	27	10	75.67	128	132.3	1.99	24.46	>100	6.28	44.1	16.8	65	33	33.76	99.5	32	999	1
95	0	93	1	1	0	0	0	25	4	74	150	495.3	13.71	7.92	0.87	2.13	41.5	15.8	27	52	14.1	45.2	3	284	1
96	1	77	1	1	1	0	0	23	10	62.67	90	182.5	9.12	141.5	1.1	7.2	38.1	13.8	11	10	3.3	171	13	3325	1
97	1	78	1	0	0	0	1	30	15	83.33	60	190.7	17.95	8.68	2.9	20	67.4	42.5	8776	4239	44.7	277.5	15	2314	1
98	1	73	1	0	1	1	0	22	9	113.6	90	192	8.5	107.6	1.26	2.98	48.4	12.4	16	16	4.98	558.8	26	204	1
99	0	86	1	1	0	0	0	19	5	65.33	82	394.3	18.2	37.82	17	2.17	143.4	30	77	38	4.9	49	15	481	1
100	0	85	1	1	0	1	0	30	10	98	112	106.5	8.71	18.14	1.63	8.63	40.1	16.8	1164	464	8.96	554.8	10	4885	1
101	1	92	1	1	1	0	1	12	0	89	96	571.2	7.84	18.45	0.05	1.3	40.2	12.9	13	9	11.3	59.7	7	153	1

102	1	72	1	1	1	0	0	24	8	83.67	142	243.8	10.04	67.23	1.77	3.23	48	19.8	37	68	35.4	96.6	3	2586	1
103	1	82	0	0	1	0	0	33	12	65	130	195.7	10.33	19	19.83	6.73	36	16.7	13	20	11.82	118.2	12	134	0
104	1	63	0	0	1	0	1	26	6	136	102	247.6	6.74	180	22.38	1.56	54.7	15.2	32	16	8.27	311.5	12	632	0
105	1	66	1	1	0	0	0	11	3	128	122	265	11.91	27	0.1	0.43	37.2	13.8	31	39	8.84	81.3	22	27	0
106	1	82	1	0	1	0	0	16	6	91	101	182	9.6	210	8.96	0.69	32.2	13.4	9	9	7.32	68.9	17	33	0
107	1	84	0	0	0	0	0	16	10	70.3	105	126.8	3.3	206	50	4.86	75.9	18.5	73	278	16.87	165.6	83	2526	0
108	1	35	0	0	0	0	0	6	4	109	96	166.6	4.74	17	0.1	1.07	37.1	14.2	147	193	20.58	60.6	16	16	0
109	1	93	1	0	0	0	0	9	3	101	115	273	10.55	160	50	2.98	38.7	17.9	14	31	5.2	91.1	13	180	0
110	1	72	0	0	0	1	1	17	6	90	100	329.3	9.2	136	141.6	1.52	44.6	22.6	19	25	4.9	198.5	10	33	0
111	1	78	1	0	1	0	0	26	5	105.7	98	148.3	4.51	43	2.75	13.87	30	16.5	65	64	7.83	125.7	72	604	0
112	1	85	1	0	1	0	0	12	5	87	78	281.4	5.78	19	0.18	1.97	37.7	15	2	11	8.48	79.4	4	343	0
113	1	72	1	1	0	0	0	25	6	110	185	394	15.9	99	0.7	3.8	64.7	16.7	60	107	18.4	92.1	15	1178	0
114	1	76	1	0	1	1	0	17	5	77.7	93		11.44	180	1.5	1.83	41.4	14.5	43	33	12.08	150.2	12	285	0
115	1	90	1	1	1	0	0	18	8	93	81	22.9	2.93	230	0.18	10.7	45.2	17.1	11	18	8.64	63.7	18	444	0
116	1	72	0	0	1		1	19	8	110	114	192	7.93	22	0.24	2.66	37	14.4	19	22	8.46	65.1	3	76	0
117	1	51	1	0	0	0	0	26	11	61.8	115	131	7.67	220	28.44	2.31	51.9	16.4	29	22	30.94	441.4	27	185	0
118	0	87	0	1	0	0	0	29	9	77.7	70	203.6	8.9	32	1.66	6.51	48.5	17.5	52	31	4.54	466.7	3	1052	0
119	1	90	1	0	0	1	0	17	5	80.7	93	310	1.86	55	8.87	2.04	47.7	16.8	24	23	11.26	243.6	4	2523	0
120	1	81	1	1	0	1	1	16	5	112	130	373.2	5.29	7	0.17	2.54	34.1	12.2	26	32	9.72	148.7	15	925	0
121	1	77	1	1	0	1	0	11	5	100.6	94	292.7	7.45	20	6.29	20	40.1	15.4	8	22	8.32	54.5	7	115	0
122	1	90	1	1	1	0	0	18	4	91.7	132	311.7	15.47	82	0.84	2	36	16.8	25	16	10.38	55.6	8	227	0
123	0	75	0	0	0	0	1	12	5	75	80	280	9.92	10	3	2.94	33.4	16.7	24	35	192	77	26	920	0
124	1	55	0	0	0	0	0	27	14	55	57	174	20.42	200	6.57	1,04	36.6	13.3	22	26	32.2	53.1	3	232	0
125	1	76	1	0	1	1	0	13	5	74	93	300	11.44	180	1.5	1.83	41.4	14.5	43	33	12.08	15.2	12	285	0
126	1	33	0	0	0	0	0	25	8	62.6	110	93.3	27.27	240	1.84	0.78	42	15.7	43	17	8.02	76	3	22	0
127	1	83	1	0	0	1	0	26	12	73	64	384	7.87	17	2.44	7.09	42.9	17.6	25	20	12.27	693.8	3	318	0
128	0	27	0	0	0	0	0	17	13	40	110	212.2	4.1	88	47.8	2.09	62.4	19.9	41	57	362.4	97.3	4	55	0
129	0	77	0	0	0	0	0	21	14	61	101	130	22.15	290	50	20	38.4	22.1	84	192	38.8	170.3	31	1354	0
130	1	81	1	0	1	0	0	20	6	125	88	191.3	12.69	175	10.61	3.38	51.1	14.8	14	18	4.72	87.5	16	434	0
131	1	89	1	1	1	1	0	10	2	102	90	315.2	6.9	87	0.18	1.12	36.1	13.7	10	13	4.04	115.2	13	121	0
132	1	71	1	0	0	0	0	6	3	90	98	136.2	8.32	190	0.15	1.44	44	16.4	73	32	6.04	54.5	14	84	0
133	1	52	0	1	0	0	0	15	7	99	119	238.3	7.1	170	344.8	16.27	74.7	18.5	221	520	16.93	282	18	202	0
134	1	87	0	1	0	0	0	22	10	46	77	494	6.4	14	0.24	2.24	45.3	16.3	55	44	20.84	149	13	781	0
135	1	77	0	0	0	0	0	20	9	92	79	221.6	15.15	250	2.9	2.83	38.4	17.5	17	17	14.03	91.1	6	268	0
136	0	50	0	0	1	0	0	21	11	91	150	309.8	17.75	240	343.4	16.37	40.5	17.6	400	709	22.28	119.4	4	218	0
137	1	90	0	0	1	0	0	17	5	78	122	406	0.4	240	44.19	1.53	43.2	16.1	107	132	144.4	92.2	7	120	0
138	0	63	1	1	0	0	1	21	9	75	60	241.4	9.22	13	0.1	1.97	37	17.8	60	41	20.99	94.2	11	1058	0
139	1	67	1	0	1	0	0	13	7	78	88	145.1	6.67	160	11.15	4	50	16.5	39	32	72.07	123	8	362	0
140	0	81	1	0	1	0	1	15	5	77	81	123.5	21.9	44	1.1	0.74	37.3	14.3	139	36	6.17	97.3	17	188	0
141	0	74	1	0	1	0	0	9	2	100	89	126	10.37	5	0.18	2.52	30	13.1	14	27	7.23	55.7	16	286	0
142	0	68	1	0	0	0	0	12	3	102	72	235.4	4.34	9	0.18	1.61	35	13	26	36	4.42	924.9	3	169	0
143	1	80	0	0	0	0	0	20	4	111.3	132	1065	8.6	47	0.9	7.03	39.4	15	22	32	6.02	61.6	21	190	0
144	1	63	1	0	0	0	0	35	14	67	129	238.3	31.26	71	2.06	0.63	143.8	21.4	40	364	16.3	119.3	10	210	0
145	1	77	1	1	0	0	1	24	10	70	92	81.5	18.68	72	50	3.4	52.3	19.8	1462	2953	12.53	285.9	126	3482	0
146	0	93	1	1	0	0	0	17	5	76	102	177.8	16.8	165	1.24	7.71	27.7	13.2	7	16	5.21	30	7	426	0
147	0	40	0	0	0	0	0	5	2	92	96	303.6	10.81	17	0.55	2.22	35.2	14.4	20	56	5.42	69.3	20	1310	0
148	1	82	0	1	1	0	0	24	12	113	105	163.6	9.7	28.14	0.25	0.32	79	24.9	11	18	5.54	49.8	11	313	0
149	1	84	1	1	0	0	1	15	4	95	94	405	13.74	74	0.12	0.87	39.5	13.5	36	28	4.61	59.4	13	376	0
150	0	87	1	0	1	0	0	30	13	87	105	356	22.57	190	104.9	10.72	37.8	15.1	119	295	48.1	211.5	114	777	0
151	1	69	1	0	0	0	0	29	10	94	79	254	19.54	38	12.19	11.88	44.7	20.2	122	278	28.6	423.5	2	72	0
152	1	83	1	1	0	1	0	9	9	83	60	739.4	7.3	18	9.54	8.05	70.1	35.4	576	944	53.06	263.9	23	1341	0

153	1	48	0	0	0	0	0	3	4	92.3	71	125	4.76	127	2.78	2.22	45.7	13.5	40	129	11.8	75.4	30	13	0
154	0	79	1	1	0	1	0	15	6	112	105	400	14.14	24	4.12	1.36	39.2	14.5	81	79	19.42	668.2	15	481	0
155	0	83	1	1	0	0	1	11	9	90.6	98	214	11.19	88	12.3	3.87	50.1	17.1	42	61	11.1	58.2	43	221	0
156	1	64	1	0	0	0	0	14	5	105	80	344	19.68	3	0.77	1.64	37	15	146	60	8.06	149	19	54	0
157	1	73	1	0	0	0	0	27	14	91.6	110	106.8	4.07	240	50	5.25	58.9	15.2	76	191	10.85	335.3	26	84	0
158	1	89	0	1	0	0	0	21	8	119.3	112	338	14.9	40	1.58	1.66	39.4	15.7	18	25	13.85	93.7	15	156	0
159	1	86	1	0	0	0	0	20	6	105	128	102	9.4	112	1.62	1.24	57	15.5	53	104	9.13	65.1	10	466	0
160	1	71	1	1	1	0	0	18	5	76.3	63	702.4	9.95	71	0.44	18.53	33	14.6	18	88	9.03	62.6	21	192	0
161	1	82	1	0	0	0	1	22	7	116	190	155.7	8.14	210	24.92	19.46	49.6	15.7	71	174	10.37	271.9	17	274	0
162	1	77	1	1	1	1	0	25	13	82.6	66		8.52	22	0.65	7.03	180	51.3	350	17	42.78	295.1	17	198	0
163	1	93	0	1	1	0	0	20	10	80	62	231.5	6.84	15	1.02	2.76	41.5	15.2	21	39	13.5	137.8	9	347	0
164	1	67	1	1	0	0	0	24	15	94	131	87.1	3.39	137	15.15	3.31	50.9	13.4	63	140	5.07	111.3	30	1370	0
165	1	75	0	0	0	0	0	9	5	76	105	213	5.97	240	1.57	3.92	37	13.8	32	43	19.93	68.7	3	96	0
166	1	83	1	0	1	0	0	12	4	89.3	94	292.7	12.77	94	4.51	2.04	42.6	15.8	42	37	18.6	185.5	5	1118	0
167	1	80	0	1	0	0	1	20	9	69	64	155.6	22.29	238	0.49	2.38	52.7	14.7	34	34	6.45	65.4	19	231	0
168	1	65	1	0	0	1	0	23	9	92	114	158.6	5.23	210	50	5.19	42.4	16.1	20	25	5.21	263.1	8	289	0
169	1	94	0	1	0	0	0	17	7	76.6	78	243.3	7.36	190	5.07	2.26	44.5	16.9	17	26	7.6	131.4	3	178	0
170	1	67	0	0	0	0	0	22	12	100.6	108	290	4.77	45	320	2.62	64.6	24.7	33	45	18.59	136.4	5	174	0
171	1	79	1	0	0	1	1	14	8	90	70	258	6.15	41	6.41	2.57	35.5	15.8	659	144	5.79	242.4	13	3320	0
172	0	93	1	1	0	0	0	27	10	63	145	126.8	14	2	0.92	1.87	36	15.9	19	29	9.85	57.7	11	118	0
173	1	72	1	0	1	0	0	6	5	80	115	191.7	7.97	11	0.22	2.73	30.1	16	45	243	40	79.8	6	125	0
174	1	71	1	0	0	0	0	25	12	100	64		9.4	23	0.06	18.99	52.7	14.5	13	33	26.16	72.2	55	658	0
175	1	67	1	0	0	1	1	28	14	82	115	257.5	22.12	37	3.55	1.52	42.6	15.4	23	18	5.07	642.9	3	467	0
176	1	65	1	0	1	1	0	28	7	95	60	445	8.33	32	0.25	5.6	37.6	14.1	10	25	40.46	400.7	10	370	0
177	0	31	0	0	0	0	0	10	3	73.6	149	198	7.53	141	41.89	20	50.8	18.5	409	656	12.79	53.1	3	67	0
178	1	78	1	1	1	1	1	13	8	104.6	91	61.1	9.73	42	0.1	1.7	32.5	14.1	17	16	7.12	460.5	39	65	0
179	0	30	0	0	0	0	0	12	5	63.6	118	339	1.54	42	0.75	2.89	42.7	18.9	13	22	12.7	57.7	3	68	0
180	1	71	1	1	0	0	0	7	0	76.3	56	440	6.32	4	1.96	6.77	40.8	16.9	61	40	10.97	88.3	7	222	0
181	1	62	0	0	0	0	0	4	3	89	90	341.5	5	6	0.16	0.22	33.4	14.5	64	371	7.79	73.8	32	317	0
182	1	74	0	0	1	0	0	12	8	49.6	105	200	2.79	48	1.58	2.75	34.6	13.1	17	15	16.7	55.5	3	200	0
183	0	62	1	0	0	0	0	25	4	132	100	338.1	12.52	120	1.2	1.93	54	19.6	14	20	5.87	31.9	16	108	0
184	1	86	1	1	1	0	0	30	12	76	108	182	25.92	57	1.49	13.8	26.1	24.3	20	14	8.96	136.8	9	194	0
185	1	84	1	0	0	0	1	16	7	70	76	164.6	5.52	108	0.27	2.81	50.8	18	12	22	8.41	54.8	12	157	0
186	0	70	0	0	0	0	0	16	5	82.6	96		9.05	62	0.44	2.9	42.4	14.6	41	48	7.95	40	9	17	0
187	0	55	1	0	0	1	0	13	5	106	89		9.2	6	3.06	1.69	33.6	13.5	35	46	1.7	839.9	26	4917	0
188	1	74	1	1	0	0	0	10	4	106	76	181.2	9	141	0.89	0.42	41.5	15.8	12	16	7	46.8	51	95	0
189	0	50	0	0	0	0	0	25	8	100	89	550	12.72	79	0.68	20	33.4	19.1	61	85	12.61	74.9	3	5	0
190	1	68	1	0	1	0	0	24	10	69.3	105	167.8	18.5	33	0.32	2.6	50	15.4	27	26	14.25	60.9	14	53	0
191	1	75	1	1	1	1	0	26	15	68.3	69	208.5	9.4	60	0.76	2.4	32.4	18.2	167	84	23.59	618.2	7	4953	0
192	0	77	1	0	1	0	0	12	2	110	80	474.1	5.93	75	0.67	20	25.6	15	18	24	25.49	68.7	8	106	0
193	0	63	0	0	1	0	0	20	15	88.3	96	172	11.33	67	5.49	20	36.1	18.8	14	65	18.86	202.4	34	706	0
194	0	71	0	0	1	1	0	11	8	81	75	428.5	9.71	278.1	242.3	16.99	57.5	15.3	31	48	26.03	400.6	12	1684	0
195	0	70	0	0	0	0	0	13	7	89	183	172.2	12.2	75.95	0.21	2.86	41.4	14.5	24	33	10.9	40.2	13	697	0
196	0	41	0	0	0	0	0	8	6	85	86	180.2	7.3	56.99	0.62	2.66	29.7	13.5	25	22	10.99	37.7	15	35	0
197	1	73	0	1	1	1	0	10	6	119	103	298.2	11.23	15	0.54	14.15	48.9	23.9	2001	5033	39.06	203.2	14	2544	0
198	1	73	1	0	0	0	0	26	12	118	132	515	15.16	10.21	14.75	20	46.7	27.5	1460	2490	154.5	143.9	2.7	1420	0
199	1	60	1	0	1	0	1	20	13	70.6	118	306.4	7.99	79.64	1.29	2.73	91	38.5	39	34	85.24	44.7	8	142	0
200	1	67	1	0	1	1	0	12	4	93	90	481	26	106	50	2.41	35.6	14.7	4	13	2.38	1170	16	3200	0
201	1	93	1	1	0	1	1	20	6	131	65	235.6	7.39	58.98	0.14	5.42	41.2	17.8	9	14	3.31	346	8	835	0
202	0	73	0	0	0	0	0	23	10	84	98	125.8	10.2	34.14	0.21	4.14	32.8	14.1	32	21	9.54	111.8	12	1589	0
203	1	77	1	1	1	0	1	21	10	93.6	92	181.8	8.02	37.36	0.42	3.42	36.2	14.9	14	30	19.02	84	18	241	0

204	1	73	1	0	1	1	0	13	7	106	55	321	9.92	22	0.18	5.72	33.5	13.2	16	21	2.68	780.7	9	433	0
205	1	67	1	0	0	0	0	27	14	47	99	113.8	6.08	66.16	2.62	6.85	91.4	30.8	52	130	210.9	296.7	36	251	0
206	1	88	1	1	1	0	0	20	6	98.6	117	427.1	13.92	155.8	3.09	6.29	30.8	14.8	21	25	19.82	91.7	3	875	0
207	1	85	1	0	0	0	0	14	6	69.3	73	255.3	7.5	55.19	0.1	2.12	43.3	13.1	43	32	2.4	78.1	26	132	0
208	1	75	1	0	1	1	0	11	8	113.6	107	155.3	16.63	42.55	0.3	1.05	48.3	15.8	31	23	8.68	109.9	3	99	0
209	0	44	0	0	0	0	0	24	15	91.6	81	194	4.06	175.9	1.66	3.47	58.4	18.3	50	31	23.17	39.6	65	164	0
210	1	65	1	0	1	1	0	22	12	95	79	378.6	15.9	212.8	50	2.51	43.9	16.1	15	16	3.44	371.6	10	223	0
211	1	74	1	0	0	0	0	16	10	73	77	242.8	5.18	240.7	61.8	3.67	40	16.1	26	18	8.29	66.9	19	256	0
212	1	68	1	0	1	1	0	22	7	74	98	410.5	6.41	204	0.5	3.95	17.7	16.9	19	25	31.8	136.1	10	132	0
213	1	62	0	0	0	0	0	33	10	112	135	221.4	24.04	118.5	1.48	12.05	59.7	18.8	31	55	7.89	36.9	19	274	0
214	1	80	0	1	0	0	1	21	9	81.3	83	236.7	17.89	22	6.46	0.82	53.2	15.5	27	24	8	100	13	115	0
215	1	42	1	0	0	0	0	17	9	86.3	70	405.9	12.38	86.22	1.24	2.04	81.9	17.1	310	169	13.78	127.2	19	981	0
216	1	71	0	0	0	0	0	26	14	109.3	85	231.3	1.06	23.02	313.2	2.83	42.2	15.3	30	31	18.22	86.7	13	102	0
217	1	42	0	0	0	0	0	22	7	79	135	1496	11.28	33.28	0.1	6.19	41.2	15.7	29	37	12.85	69.5	18	24	0
218	1	82	0	1	0	0	0	25	9	86	146		49.86	182.2	0.29	0.85	60.3	17.2	54	44	8.84	76.2	12	326	0
219	1	75	1	1	1	0	0	14	7	64.7	104	244.4	9.85	276.7	65.59	1.42	85.8	48.9	5	25	21.97	167.2	9	2272	0
220	1	84	0	0	0	0	0	24	10	95.3	122	118	14.92	17.36	0.11	5.45	37.5	19.5	389	326	63.79	90.6	9	665	0
221	1	69	0	0	0	0	0	16	9	99.6	67	263.2	13.83	0.5	0.1	3.45	36.2	14.8	30	30	20.06	98.4	8	418	0
222	1	94	0	1	0	0	0	21	13	68	115	384.3	13.63	2.79	0.1	4.82	52.1	17.3	24	31	7.19	126.8	5	99	0
223	0	73	1	0	1	1	0	23	11	103	87		19.18	25.52	0.1	5.01	37.8	13.7	44	41	20.2	69.9	6	810	0
224	0	82	0	1	0	0	1	20	8	94	69	413.8	13.07	6.62	0.1	1.14	35.6	15	33	36	18.74	130.9	10	766	0
225	1	80	0	0	0	0	0	28	12	87	133	128.8	11.38	221.5	0.88	20	38.1	17.9	24	17	18.5	119	1	88	0
226	1	71	0	1	0	0	0	30	11	65	110	140.3	7.19	329.8	41.07	2.28	50.3	16.6	323	272	19.4	481.9	36	200	0
227	0	75	1	0	0	0	0	23	11	112.6	137	189.2	12.01	264.9	141.3	5.36	37.6	19.9	186	140	49.2	103.3	8	658	0
228	1	59	1	0	1	1	0	14	6	106	92	333.3	13.23	253.5	60.07	11.61	76.6	23.2	2999	3158	32.8	703.8	79	1922	0
229	1	81	1	0	0	0	0	23	7	126.6	90	391	26.85	44.36	0.87	1.69	37.2	14.7	375	105	32.84	89.3	3	135	0
230	0	86	1	0	1	0	0	21	10	72	98	305.2	7.64	24.78	0.12	1.84	46.8	19.5	3	20	19.26	270.2	8	1349	0
231	1	61	0	0	0	0	0	21	6	118.6	53	264.3	19.67	8.16	0.65	15.49	37.5	12.7	20	70	4.98	96.6	5	117	0
232	1	52	1	0	0	0	0	15	9	88	47	432.7	26.55	2.25	1.48	1.25	46.6	14.8	364	750	48.3	807.2	478	492	0
233	1	42	0	0	0	0	0	22	9	84	136	316.3	22.17	38.52	1.04	9.16	36.4	14.3	40	73	24.35	88	24	129	0
234	1	72	0	0	0	0	0	17	12	69	82	100.4	7.18	0.5	0.1	4.02	50	16.4	27	22	26.1	91.1	3	14	0
235	0	59	0	1	1	0	0	17	6	66.6	100	446.8	9.8	444.7	12.39	2.63	42.2	18	20	14	14.34	60.8	8	335	0
236	0	79	0	0	0	0	0	30	14	113.8	111	266.6	12.95	52.64	0.1	20	46.2	16.4	24	33	16.2	43.1	20	78	0
237	1	78	1	1	0	1	0	28	12	93	124	171.2	8.02	33.04	0.47	2.75	45.5	14.4	22	33	27.63	231.2	14	1880	0
238	0	80	1	0	1	0	0	24	11	84.3	72	242.5	11.28	43.69	0.39	1.09	51.8	17.2	14	16	3.55	87	17	991	0
239	1	59	1	0	0	0	0	11	6	86.3	90	199.6	8.04	109.6	0.19	0.98	36.7	14.3	32	36	14.36	95.6	8	37	0
240	0	83	1	0	0	0	0	20	8	123.3	111	98	7.64	114.8	4.78	4.1	45.1	12.1	17	14	12.21	49.2	8	357	0
241	0	80	0	1	0	0	0	33	14	71	114	104.4	5.84	64.53	1.31	4.04	63.6	24.4	58	586	30.27	137.3	30	1229	0
242	1	72	1	0	0	0	0	26	13	73.3	53	328.6	7.25	80.75	0.05	2.76	41.9	14.8	10	28	13.04	129.2	23	825	0
243	0	82	1	0	1	1	0	19	9	69	56	358.8	10.38	1.02	0.1	1.5	41.6	14.7	30	22	11.1	337.2	10	573	0
244	1	68	1	0	1	1	0	31	10	90	70	215.3	10.91	21.27	66.4	2.14	33.4	15.1	19	38	3.15	688.6	29	1288	0
245	1	87	0	0	0	1	0	30	12	102.6	80	374.3	11.47	72.28	11.18	12.92	45.7	16	34	52	5.16	375	24	5.33	0
246	0	46	1	0	1	1	0	23	12	101.6	42	349	1.45	82.12	0.7	5.43	53.5	14.7	15	29	11.43	572.7	17	5021	0
247	1	85	1	1	0	0	0	18	7	91.6	74	247.8	13.04	104.4	23.99	9.38	50.1	16.3	78	249	9.74	106.6	12	1405	0
248	1	82	1	0	0	1	0	33	12	68	62	216.6	17.08	39.78	0.09	2.45	38.8	14	37	43	7.99	423.4	12	1845	0
249	1	20	1	0	0	0	0	15	7	82.6	120	511	31.63	0.85	0.17	2.2	29.1	14.2	66	32	28.4	47.6	3	224	0
250	0	50	0	0	0	0	0	12	3	94	111	240.2	5.2	177.8	18.9	2.44	41.6	15.7	14	16	9.5	38.4	6	26	0
251	0	57	0	0	0	0	0	22	11	99.7	78	243	9.44	108.5	2.66	7.97	44.8	23.7	162	738	48.4	86.8	303	520	0
252	1	69	1	1	0	0	0	39	14	59.7	110	375.4	21.45	53.82	12.34	20	180	51.6	122	173	16.2	202	21	306	0
253	1	89	0	0	1	0	0	15	7	77	105	123.7	7.67	83.91	0.1	3.4	37.1	16.1	42	38	15.67	62.2	9	257	0
254	1	53	0	0	0	0	0	18	8	98.3	108	333.3	8.01	9.6	1.57	1.6	33.4	15.1	24	26	23.78	76.5	3	264	0

255	1	63	0	0	0	1	0	36	14	52.3	126	402.7	0.08	61.85	273.1	5.2	75.5	25.3	139	642	22.3	241.4	249	159	0
256	1	62	1	0	1	1	0	23	7	110.6	70	200.2	6.51	1.21	0.23	2.31	32.8	14	60	51	51.34	427.1	30	650	0
257	0	50	1	0	0	0	0	32	9	113	118	181.7	9.39	10.64	0.32	2.35	45.5	15.1	189	266	12.67	27	3	88	0
258	0	79	1	0	0	0	0	7	5	82.6	100	186.3	4.08	0.66	0.28	11.77	34.2	14.8	11	25	12.7	54.7	1	1594	0
259	1	51	0	0	0	0	0	23	9	87	127	213.4	5.72	299.6	35.45	4.55	50.3	22.1	15	51	18.7	79.6	10	206	0
260	0	71	1	0	0	0	0	21	11	81.3	120	0.2	6.81	297.2	0.37	20	48.4	18.6	33	127	27.98	58.2	24	113	0
261	1	77	1	1	1	1	0	9	9	75.3	118	295.1	11.92	293.9	61.69	2.21	117.4	26.8	10	19	9.86	251	7	3744	0
262	1	76	0	0	0	0	0	17	10	100.6	103	376.3	9.63	151.8	35.22	1.08	48.6	15.1	38	38	34.42	77.6	22	297	0
263	1	62	1	0	1	0	0	19	7	125	104	251.8	15.1	10.26	0.14	0.6	32.7	14.3	16	19	24.04	105.8	3	128	0
264	1	75	1	0	0	0	1	11	2	111.3	79	246.9	13.99	59.65	0.05	1.53	33.8	15.6	29	14	6.7	37.9	1	174	0
265	1	25	0	0	0	0	0	16	10	77	120	222	9.32	222.3	0.05	20	41.8	17.4	12	13	10.12	52.6	8	183	0
266	1	89	1	1	1	0	0	34	11	86.3	128	127.1	19.12	268.8	18.52	1.9	34.1	14	21	19	10.8	147.3	2	714	0
267	1	63	0	0	0	0	0	21	11	61	77	102.2	4.14	38.87	0.1	2.47	38.7	16	41	55	34.69	13.3	47	174	0
268	1	84	1	0	0	0	0	23	12	75	68	531.3	12.68	160.6	6.49	5.58	37.4	17.3	151	218	36.92	149.3	48	179	0
269	1	82	1	1	1	1	0	22	8	99	65	368.1	16.17	8.98	2.1	15.15	38.7	20.6	41	56	35.57	87.9	8	984	0
270	0	28	0	0	0	0	0	6	2	72.6	96	244.2	25.84	30	0.19	2.03	37.2	17.7	45	151	3.7	28.9	184	584	0
271	1	75	0	0	1	0	0	21	6	88	128	247.3	13.92	125.2	0.13	3.09	38.9	18.1	130	108	6.69	59	15	62	0
272	1	89	0	0	1	0	0	17	4	94.3	92	312.5	14.13	121.5	0.75	2.59	37.2	15.8	7	11	12.86	39.7	3	104	0
273	0	53	0	0	0	0	0	9	3	81.3	112	400	12.25	254	9.61	20	57.1	18.7	23	69	10.01	74.3	39	337	0
274	0	67	1	0	0	0	0	19	10	80	92	140.8	3.21	78.61	0.11	5.21	37.5	15.7	40	112	19.12	22.7	12	162	0
275	1	60	0	0	0	0	0	34	11	79	120	170.5	10.7	20	0.75	5.12	47	14.9	166	159	11.76	16.8	8	65	0
276	1	91	1	0	1	0	0	39	14	31.7	135	160.2	19.03	34.38	7.42	20	73.6	29.5	61	86	21.58	344.9	16	127	0
277	1	71	0	1	1	0	0	20	10	55.3	140	205	6.29	192	0.96	3.76	50.9	18	32	52	23.98	144.8	53	101	0
278	0	46	1	0	0	0	0	21	4	87	76	443.5	20.17	35.79	0.88	20	40.3	14.5	31	34	13.88	68.7	14	427	0
279	1	96	1	0	0	0	0	24	7	61.6	87	344	13.36	62.29	0.27	2.11	38	14.8	19	25	6.22	125.6	14	55	0
280	1	53	0	0	0	0	1	19	9	118	92	378	7.01	2.07	0.1	4.67	33.6	13.8	63	42	7.31	37.6	9	302	0
281	1	89	0	0	1	0	0	25	13	103.3	110	200	5.51	42.16	0.1	3.93	38.9	14.1	10	11	11.16	70.3	6	169	0
282	0	34	0	0	0	0	0	20	8	53.6	108	425.7	18.19	0.5	67.69	20	180	36.3	681	1101	19.16	104.1	81	49	0
283	1	52	1	0	1	0	0	19	13	105	85	93.1	8.94	22.6	1.39	7.29	52.7	22.9	367	690	11.12	500.7	77	1644	0
284	1	68	0	0	0	0	0	12	4	74	122	393	25.69	136	3.67	1.81	45	16.6	52	89	39.34	116.9	8	362	0
285	0	80	1	1	0	1	0	13	7	94.6	121	220.8	5.44	104.8	3.79	4.46	34.2	15.2	10	27	5.53	215.8	15	1131	0
286	1	55	0	0	1	0	0	17	8	89	87	203	11.7	170	0.32	1.42	36.8	14	18	32	27.55	52.3	11	147	0
287	0	79	1	1	0	0	0	15	7	120.3	45	302.4	10.94	14.73	0.11	1.43	39.9	16.3	38	256	13.3	102.1	98	1820	0
288	1	98	1	1	0	0	0	27	11	77.7	89	221.8	8.71	121.1	0.53	2.92	59.7	15.6	27	19	4.08	862	6	244	0
289	1	84	1	0	0	0	0	21	8	85.6	46	107.8	6.7	4.27	0.15	1.42	35.1	15.5	87	68	26.86	118.9	3	1289	0
290	0	83	0	0	1	1	0	15	7	93.3	95	235.2	19.61	90.37	0.06	3.03	31.1	12.6	10	17	7.28	66.1	5	170	0
291	1	74	0	0	0	0	0	23	13	76	90	128.3	14.6	199	0.64	6.68	38.3	17.2	91	105	34.98	85.2	4	96	0
292	1	70	1	0	1	0	0	16	8	130	98	240.7	12.83	0.5	0.1	0.44	42.5	19.6	17	35	15.83	195.2	22	139	0
293	1	87	0	0	0	0	0	26	12	74	85	172.4	4.08	46.99	0.1	11.6	36.4	13.8	15	106	12.1	27.9	4	195	0
294	1	82	1	1	0	0	0	43	16	74.9	80	127	9.46	6.77	0.09	2.39	31.4	13.6	23	33	4.7	86.3	27	133	0
295	1	26	1	0	0	1	0	16	7	84.7	73	90.8	9.66	106.2	0.42	1.42	42.7	15	48	48	2.17	92.8	65	63	0
296	1	70	0	0	0	0	0	24	10	95.7	110	189.4	0.64	118.7	44.85	4.13	42.8	17.7	9	66	42.8	83	39	1818	0
297	1	96	1	0	0	0	0	22	11	75.3	79	238.3	8.42	2.64	0.05	1	38.8	14.1	58	38	2.94	75.7	10	286	0
298	1	77	0	1	0	0	0	28	12	58.6	93	238.1	13.96	209.2	1.48	20	38.5	16.7	10	27	12.6	66.7	9	94	0
299	0	64	1	0	0	0	0	19	10	45	83	410.5	6.53	32.33	0.1	1.15	41.1	14.6	7	18	5.67	60.6	3	141	0
300	0	82	1	1	0	0	0	16	8	75.3	80	244.3	5.24	12.87	0.1	20	27.5	12.6	8	25	13.6	33.7	21	453	0
301	0	81	1	0	0	0	0	17	7	80.7	80	522.5	15.71	21.09	0.24	4.04	40.7	15.6	9	14	31.87	138.9	3	147	0
302	1	68	1	1	1	0	0	7	2	106	81	316.4	10.17	0.5	0.05	0.41	37.5	13.9	33	46	44.43	54.6	3	42	0
303	1	87	1	1	0	1	0	22	11	90.3	84	128.3	5.19	122.3	0.61	1.4	48	17.3	9	14	2.59	381.9	3	212	0
304	1	88	1	0	0	0	0	18	6	87	83	161	16.72	72.16	0.44	7.27	35.5	14.4	67	39	7.74	72.1	11	214	0
305	1	63	1	0	0	0	0	14	6	105.3	70	183.6	16.76	14.73	0.77	1.67	35.1	14	16	31	20.72	62.3	7	195	0

306	0	70	1	1	1	0	0	18	6	82	87	318	11.21	115.9	0.81	18.25	48.2	16.6	14	19	9.58	78.3	12	142	0
307	1	82	1	1	1	0	0	25	13	83	97	78.2	15.85	122.7	0.33	8.47	35.6	13	32	61	10.12	193.6	34	67	0
308	0	31	0	0	0	0	0	26	10	89	148	136.6	11.23	13.27	0.05	1.6	45.5	13.3	146	99	5.33	28.6	21	65	0
309	0	76	1	1	1	0	0	22	7	88	81	80.3	5.23	83.23	0.1	1.11	39.8	16.6	30	23	6.15	29.7	5	1008	0
310	0	74	1	1	1	1	0	25	10	106	81	152.2	17.82	105.1	0.16	2.08	45.5	15.6	9	11	3.56	358.7	3	207	0
311	1	86	0	1	1	1	1	14	6	90.6	109	275.1	6.81	167.8	0.11	1.46	43.9	14.8	16	15	3.61	130.3	15	304	0
312	1	62	0	0	0	0	0	15	5	71	109	285.7	8.96	86.14	0.1	4.3	51.1	14.3	50	56	7.08	38.8	16	414	0
313	0	66	1	0	1	1	0	22	8	136	110	392.4	17.95	3.61	0.68	0.74	24.2	13.5	40	39	6.47	709.5	23	4964	0
314	1	56	1	0	0	0	1	14	4	91	114	221	7.19	10.32	0.05	0.87	37.5	14.5	33	23	6.71	53.4	4	54	0
315	0	59	0	0	1	0	0	29	9	64	110	131	35.97	24.61	0.06	2.01	35.5	13.4	20	44	10.7	23.9	419	229	0
316	1	81	1	0	1	0	0	25	6	76.67	60	285.5	8.61	7.49		0.68	39.3	13.9	12	7	1.34	27.5	3.4	23.4	0
317	1	91	0	1	0	0	0	22	4	95	118	252	6.29	28.24	0.12	1.63	56	17.4	36	17	3.62	63.4	1.1	96.1	0
318	0	91	1	0	0	0	0	22	4	95.67	86	312.9	9.92	12.92	0.05	1.16	36.7	13.8	35	10	3.19	69.7	6	103	0
319	1	81	1	1	0	0	0	20	8	88.67	85	131.8	8.17	3.8	0.05	1.27	47	15.3	22	29	6.75	90.9	5	120	0
320	1	66	0	0	1	0	0	25	6	69.69	79	201.5	6.53	3.73	0.05	0.95	44.6	15.4	43	144	10	25.1	6	65	0
321	1	64	1	0	0	0	0	20	5	96.33	77	360.5	8.26	32.42	0.05	1.49	34.1	13.8	13	10	6.89	25.4			0
322	1	72	1	0	1	0	1	23	10	71.67	75	170.2	8.84	16.06	1.67	2.02	54.9	21	22	9	13.26	219.3	1	903	0
323	1	85	0	0	0	0	1	12	2	77.33	74	348	12.55	36.89	1.09	0.95	50.5	21.9	62	63	10.9	59	6	331	0
324	1	92	1	0	0	0	0	16	4	84.67	101	374.5	4.79	10.62	0.73	18.85	35.4	15.6	229	300	11.8	107.3	3	300	0
325	1	57	1	0	1	1	0	15	4	106.6	82	585.2	11.31	22.82	0.1	7.42	50.7	14.6	7	12	16.4	473.3	8	124	0
326	0	25	0	0	0	0	0	7	0	86.67	82	457	5.22	0.92	0.05	0.81	31.9	12.6	26	47	5.82	29.8	10	33	0
327	1	45	1	0	0	1	0	24	10	72	64	318.2	2.19	70.88		5.98	48.1	16	13	14	6.05	869.6	13	151	0
328	1	70	1	0	1	0	0	28	9	80.67	61	320.7	23.21	170.3	2.53	3.6	58.2	14.8	18	41	33.7	1171	3	241	0
329	0	66	1	0	0	0	0	23	5	120.6	97	355.8	10.59	1.99	0.05	1.87	30.3	13.2	29	34	7.7	39.2	20	27	0
330	1	66	1	0	0	1	0	34	9	63	74	318.2	15.11	11.53	0.97	4.42	77.1	52	154	69	7.52	260.3	38	1304	0
331	1	81	1	1	0	1	0	25	9	66	128	291.6	15.22	159.2	92.71	4.06	38.7	16.1	102	36	21.4	263	50	225	0
332	1	62	1	0	0	0	0	24	14	77.33	136	179.2	1.08	232.9	1.85	0.97	38.6	17.1	25	19	88.5	92.6	3	358	0
333	1	74	0	0	0	0	0	33	16	97.33	83	101.7	0.08	256.8	41.71	20	81.3	36.7	642	1731	51.4	276.2	56	2793	0
334	0	87	1	0	1	0	0	24	6	75	75	107.5	16.86	232.5	0.6	20	45.1	17.2	24	27	8.1	45.3	4	462	0
335	1	75	1	0	0	0	0	14	2	107	98	357.3	13.67	145	0.05	0.38	47.4	13.5	13	10	19.42	41	14	67	0
336	0	81	1	0	1	0	0	27	8	94.67	140	443.6	7.77	59.24	3.85	13.05	48.7	29.4	40	13	75.66	161.7	9	357	0
337	1	70	1	0	0	0	0	19	7	81.88	127	237.6	17.62	189.7	0.32	20	36.7	16.6	20	22	11.45	69.6	3	296	0
338	0	63	1	0	0	0	0	17	6	99.67	105	188.2	12.38	93.01	3.66	0.99	39.5	13.6	25	18	9.4	29.8	3	152	0
339	1	85	0	1	0	0	0	24	6	124.3	159	101	11.58	209.6	13.65	3.8	42.5	16.8	18	13	9.2	82.6	8	399	0
340	1	66	1	0	0	0	0	22	8	102.6	132	139.2	35.58	144.1	3.01	2.86	33.3	17.3	23	20	28.6	31.5	3	220	0
341	0	85	1	1	1	1	0	27	9	110.3	73	161.8	12.17	17.66	0.07	12.71	34.1	14.2	24	19	6.1	330.3	7	1360	0
342	1	83	1	0	0	0	0	30	10	114.3	86	131.8	10.24	79.88	49.66	6.93	40.9	17.1	28	10	22.53	184.6	31	844	0
343	1	73	1	0	1	0	0	19	10	72.67	70	83.22	11.13	127.8	1.95	3.6	33.7	16.5	14	19	7.1	438.2	11	970	0
344	1	55	0	0	1	0	0	14	8	58.67	74	228.2	10.55	0.5	0.5	1.4	41.6	15.5	15	17	23.91	42.4	3	29	0
345	0	57	1	0	0	0	0	30	16	56.67	93	198.1	22.75	43.45	70.49	20	98.2	30.9	3012	1993	56.32	262	59	1118	0
346	0	81	0	0	0	0	0	23	9	102	61	424.6	14.38	120.6	3.05	2.29	62.6	15.9	65	59	17.6	201.5	26	384	0
347	0	30	0	0	0	1	0	9	3	79.33	76	307.2	7.95	15.25	0.05	1.26	52.3	30.4	18	12	14.4	109.2	3	222	0
348	0	38	0	0	1	0	0	10	3	104.6	118	329.5	7.48	206	38.04	5.39	36.3	14.1	727	552	21.74	83.4	8	123	0
349	1	52	1	0	0	0	0	13	6	90	101	254.6	6.45	57.53	0.49	6.9	46.3	13.9	31	14	8.62	1454	55	63	0
350	1	72	1	1	1	0	1	31	14	57.67	139	62.7	10.82	10.68	2.2	4.11	68.1	20.1	49	15	72.36	189.2	41	548	0
351	0	24	0	0	0	0	0	19	7	91.33	101	184.8	14.97	0.5	0.05	3.65	31.5	14.3	47	28	13.2	54.6	14	35	0
352	0	79	0	0	0	0	0	31	11	52	71	365.5	1.84	39.37	27.28	3.55	87.9	22.8	66	39	17.8	134.3	24	151	0
353	0	58	1	1	1	1	0	16	6	82.67	79	208.1	3.82	44.07	3.22	2.78	47.2	15.9	14	<6	4.2	414	3	1481	0
354	1	90	0	0	1	0	1	17	6	77.67	69	166.3	6.59	5.51	0.05	0.82	41.1	14.4	36	42	6.76	35.8	10	75	0
355	1	60	0	1	1	0	1	12	4	92.33	87	239.3	8.68	26.57	9.93	5.02	35	15.2	112	197	13.41	70.5	12	602	0
356	1	71	1	0	0	0	1	16	8	96.33	84	168.4	11.29	5.78	1.51	2.66	40.4	14.6	37	33	25.1	82	8	152	0

357	1	78	0	1	0	0	1	29	13	104.3	120	213.6	14.18	84.77	65.47	20	44.8	18.3	532	799	26.53	357.4	49	2929	0
358	1	76	1	0	0	1	0	20	8	96	89	260	9.89	51.85	1.35	1.39	42.9	15.6	38	20	7.6	811.7	7	69	0
359	0	71	0	0	0	0	1	14	4	66.67	82	245.5	5.39	0.5	0.05	20	32.4	14.2	22	15	9.4	40.7	7	121	0
360	1	87	1	1	1	0	0	24	8	67.67	110	97.14	18.04	229.4	6.58	1.5	55.4	16.8	34	37	7.4	123.7	17	279	0
361	1	74	0	0	0	0	0	33	12	75	136	53.1	29.55	110	26.53	6.24	41.7	18.1	19	12	29.6	118.1	12	216	0
362	1	56	0	0	0	0	0	27	14	95.33	149	57.9	0.66	>360	81.9	3.17	45.8	13	69	33	94.9	103	11	303	0
363	0	71	1	0	1	0	0	23	6	83.67	102	318.8	22.33	147.7	17.88	5.17	33.9	16.5	40	28	4.79	930.2	12	114	0
364	1	58	0	0	1	0	0	31	7	49.33	151	435.8	18.14	0.5	0.83	2.32	36.9	15.4	44	50	26.2	65.3	4	94	0
365	1	53	1	0	0	0	0	2	0	100	69	403.9	6.11	0.5	0.13	1.96	31.6	14.6	29	22	16.9	59.7	7		0
366	1	53	1	0	1	0	0	22	4	93.67	144	479.3	27.38	25.95	3.04	3.85	41.1	15.6	10	11	5.85	106	19	571	0
367	0	69	1	0	0	0	0	14	8	88	150	290.5	1.47	153.5	5.09	6.31	42.7	25	51	29	70.21	155.8	17	345	0
368	1	75	0	1	1	0	0	32	10	89.67	124	71	80.63	17.81	0.41	20	37.3	18.5	125	24	11.3	75.2	49	220	0
369	0	76	1	1	1	0	0	34	12	72.67	94	116.9	5.23	145.6	6.76	6.45	46.4	19.5	35	21	7.5	200.9	2	3009	0
370	1	68	0	0	1	0	1	30	6	117	153	107.6	18.43	156.6	17.12	1.73	43.5	15.6	27	21	12.5	68.5	10	312	0
371	0	70	1	0	0	0	0	34	14	102.3	140	137.6	0.16	280.1	10.58	4.03	37.8	16	34	36	25.4	51.7	8	58	0
372	0	58	1	1	1	1	0	28	8	96.67	86	195.4	7.64	137.3	0.05	1.81	46.8	14.2	12	17	6.61	851.6	16	4949	0
373	1	74	0	0	0	0	0	22	6	77	89	369.5	24.71	39.16	0.83	2.98	37	15	29	27	9.7	51.2	13	87	0
374	1	56	1	0	1	0	0	22	7	73.33	110	260.4	13.98	72.37	1.89	2.61	41.3	13.2	11	20	11.8	80.9	7	123	0
375	1	83	1	1	0	0	0	30	11	44.67	67	111.2	12.08	45.17	0.39	2.23	53.7	44	573	466	33.9	144.6	65	186	0
376	0	55	0	0	0	0	0	26	12	61.33	110	346	3.68	141.9	79.34	2.09	43.9	24.7	42	13	37.1	128.6	28	210	0
377	1	37	0	0	1	0	0	25	11	93	88	53.5	14.21	67.02	0.48	3.93	39.3	16.6	38	131	44.31	115	17	953	0
378	1	54	1	1	1	1	0	16	9	94	79	210.3	4.43	28.08	1.21	4.62	52.5	16.3	20	14	8.7	726.1	5	3360	0
379	1	87	0	0	0	0	0	18	9	100.3	92	213.8	6.1	26.1	0.49	3.03	45.8	17.1	45	12	6.36	812.5	32	919	0
380	0	84	0	0	0	0	0	34	10	56.67	136	89.83	22.81	17.52		9.01	36.9	15.5	15	16	7.28	53.5	6	785	0
381	1	81	1	0	1	0	0	35	10	91	62	70	9.85	233.7	5.23	2.62	48.9	18	95	101	23.7	119.2	11	413	0
382	0	69	0	0	0	0	0	22	9	110.6	133	255.1	15.49	13.48	1.8	18.73	48.2	14.1	63	80	24.02	62.1	20	63	0
383	0	79	1	0	0	1	0	23	7	101	91	450.6	17.56	122.1	5.86	16.61	28.7	14.4	39	30	4.73	623.5	25	220	0
384	1	68	0	0	0	0	0	16	8	125.3	79	348.1	36.45	48.85	12.49	3.59	148.8	18.1	5571	1643	235.6	203.7	488	289	0
385	1	82	1	0	0	0	1	16	2	101.6	95	375.6	5.3	0.5	0.05	1.48	34.6	13.2	48	85	8.02	96.1	22	1179	0
386	1	77	1	0	1	0	0	19	6	106.3	121	230.8	25.96	214	44.68	4.34	34.1	15.2	133	92	17.81	81.6	158	558	0
387	0	55	1	0	1	0	0	23	12	90	95	178.8	2.82	36.02	12.05	2.54	43.9	15.6	18	22	5.3	195.9	7	309	0
388	1	72	0	0	0	0	1	14	3	103.3	106	180	9.26	134.9	0.1	2.5	43.5	16	18	26	10.8	52	7	513	0
389	1	65	1	0	1	0	0	18	10	102.6	77	168	8.23	104	0.34	0.74	36.1	13.6	22	20	31.9	126.3	3	201	0
390	1	72	1	0	1	0	0	19	6	91.33	97	107.8	13.03	102.7	0.63	7.44	52.4	16.1	34	19	8.6	188.7	3	2165	0
391	0	82	0	0	0	0	0	20	7	74	93	338.1	0.3	88.95	1.22	13.23	36	17.5	20	22	54.84	98.7	5	668	0
392	0	85	0	0	0	1	0	24	7	77	93	521	6.63	20.41	0.12	2.16	20.3	12.9	22	24	8.7	225.7	14	291	0
393	0	83	1	0	0	1	0	32	11	53.33	142	447.8	9.52	275.8	50	20	45.3	15.7	71	13	15.3	584.1	5	717	0
394	0	79	1	0	1	0	0	27	7	81	119	111.3	9.84	75.19	50	9.3	40.8	13.7	61	19	7.71	90.1	31	520	0
395	1	84	1	0	1	0	0	15	3	91	102	222.9	13.08	0.5	0.58	20	40	14.3	43	33	11	49.6	13	19	0
396	1	83	1	1	1	1	0	14	5	110	102	394.2	10.04	5.02	0.07	2.54	43.5	14.1	24	19	9.2	367.5	14	4891	0
397	1	47	1	0	0	1	0	20	5	115.3	120	342.7	6.8	3.28	1.12	3.51	43.3	13.6	22	23	9.1	2128	44	96	0
398	1	52	0	0	1	0	0	19	7	101.6	116	481.6	28.78	0.5	0.5	5.82	96.4	17.2	80	39	10.4	87.5	221	5	0
399	1	72	1	0	1	0	0	22	7	84	108	101.2	31.77	157.4	2.39	1.98	51.2	17.3	55	24	5.12	145.5	12	268	0
400	1	83	1	0	1	1	0	31	6	105.3	103	469.7	7.83	139.2	0.88	2.47	46.5	12.5	13	7	1.92	379.3	23	190	0
401	1	68	1	1	1	1	0	27	8	76	99	189.3	5.87	42.36	0.07	1.69	30.7	14.3	11	12	6.64	129.2	11	231	0
402	1	83	0	1	0	0	0	31	8	90	130	173.5	23.02	52.03	0.05	1.84	44.8	14.1	27	26	6.8	189.2	5	285	0
403	1	45	1	0	0	0	0	6	2	71.67	74	407	8.88	0.5	0.5	0.56	101	14.1	19	40	12.8	61.6	5	43	0
404	0	83	1	1	1	0	0	40	9	58	133	495.5	20.85	21.8	3.35	11.65	39.5	15	256	31	9.79	172.7	48	194	0
405	0	70	0	0	1	0	0	8	5	70.67	79	381.5	11.41	2.01	3.07	0.36	180	20.5	21	14	8.26	146.9	48	857	0
406	0	40	0	0	0	0	0	1	3	105.6	74	300	6.27	0.5	0.05	0.41	36	12.5	21	23	11.6	71	14	43	0
407	1	66	0	0	0	1	1	33	12	70	91	122.9	8.94	120	12.07	7.89	45.3	16.6	1444	861	10.32	439.6	38	1890	0

408	0	84	1	0	0	0	0	26	8	86	118	175.6	34.97	113.3	11.88	4.37	37.5	13.5	33	37	17.7	374		196	0
409	1	59	1	0	1	1	0	41	10	126.3	102	460	10.44	5.94	0.24	1.33	24.6	12.2	23	24	9.8	912.9	5	337	0
410	1	77	1	1	1	1	0	25	9	73	74	272	10.83	0.74	0.12	1.37	41.9	12.7	33	8	5.23	488.6		1043	0
411	0	85	1	0	1	1	0	34	13	103.3	123	117.6	34.34	259.9	50	20	48.2	16.6	35	10	8.13	596.5	9	5009	0
412	1	81	0	0	1	0	0	20	7	82	90	425	10.01	171.2	1.78	2.41	42.1	16.4	29	23	10.39	90.1	11	351	0
413	0	43	1	0	1	1	0	15	6	109.6	104	127.8	9.75	29.65	0.18	0.7	31.4	13.1	19	19	2.46	411.6	11	1396	0
414	0	82	1	0	1	1	0	22	6	98.67	92	538.1	9.4	109.2	0.37	20	33.3	14.7	27	45	7.3	95.7	9	82	0
415	0	71	1	0	1	0	0	23	7	122.6	74	224.4	3.24	3.88	0.11	1.55	57.6	14.1	24	34	7.7	813.1	13	157	0
416	1	46	1	0	0	0	0	14	13	75.33	111	105	12.17	103.9	4.3	5.94	52.1	16.1	216	55	50.23	302.3	74	143	0
417	1	87	1	0	0	0	0	23	9	91	99	388.6	19.1	6.72	1.15	1.94	45.1	17.2	117	13	23.91	394.8	13	188	0
